# Cinaciguat (BAY-582667) Modifies Cardiopulmonary and Systemic Circulation in Chronically Hypoxic and Pulmonary Hypertensive Neonatal Lambs in the Alto Andino

**DOI:** 10.3389/fphys.2022.864010

**Published:** 2022-06-06

**Authors:** Felipe A. Beñaldo, Claudio Araya-Quijada, Germán Ebensperger, Emilio A. Herrera, Roberto V. Reyes, Fernando A. Moraga, Alexander Riquelme, Alejandro Gónzalez-Candia, Sebastián Castillo-Galán, Guillermo J. Valenzuela, María Serón-Ferré, Aníbal J. Llanos

**Affiliations:** ^1^ Laboratorio de Fisiología y Fisiopatología del Desarrollo, Programa de Fisiopatología, ICBM, Facultad de Medicina, Universidad de Chile, Santiago, Chile; ^2^ International Center for Andean Studies (INCAS), Universidad de Chile, Santiago, Chile; ^3^ Departamento de Ciencias Biomédicas, Facultad de Medicina, Universidad Católica del Norte, Coquimbo, Chile; ^4^ Institute of Health Sciences, University of O’Higgins, Rancagua, Chile; ^5^ Laboratory of Nano-Regenerative Medicine, Research and Innovation Center Biomedical (CIIB), Faculty of Medicine, University of Los Andes, Santiago, Chile; ^6^ Department of Women’s Health, Arrowhead Regional Medical Center, San Bernardino, CA, United States

**Keywords:** Cinaciguat, hypoxia, pulmonary hypertension, newborn, high altitude

## Abstract

Neonatal pulmonary hypertension (NPHT) is produced by sustained pulmonary vasoconstriction and increased vascular remodeling. Soluble guanylyl cyclase (sGC) participates in signaling pathways that induce vascular vasodilation and reduce vascular remodeling. However, when sGC is oxidized and/or loses its heme group, it does not respond to nitric oxide (NO), losing its vasodilating effects. sGC protein expression and function is reduced in hypertensive neonatal lambs. Currently, NPHT is treated with NO inhalation therapy; however, new treatments are needed for improved outcomes. We used Cinaciguat (BAY-582667), which activates oxidized and/or without heme group sGC in pulmonary hypertensive lambs studied at 3,600 m. Our study included 6 Cinaciguat-treated (35 ug kg^−1^ day^−1^ *x* 7 days) and 6 Control neonates. We measured acute and chronic basal cardiovascular variables in pulmonary and systemic circulation, cardiovascular variables during a superimposed episode of acute hypoxia, remodeling of pulmonary arteries and changes in the right ventricle weight, vasoactive functions in small pulmonary arteries, and expression of NO-sGC-cGMP signaling pathway proteins involved in vasodilation. We observed a decrease in pulmonary arterial pressure and vascular resistance during the acute treatment. In contrast, the pulmonary pressure did not change in the chronic study due to increased cardiac output, resulting in lower pulmonary vascular resistance in the last 2 days of chronic study. The latter may have had a role in decreasing right ventricular hypertrophy, although the direct effect of Cinaciguat on the heart should also be considered. During acute hypoxia, the pulmonary vascular resistance remained low compared to the Control lambs. We observed a higher lung artery density, accompanied by reduced smooth muscle and adventitia layers in the pulmonary arteries. Additionally, vasodilator function was increased, and vasoconstrictor function was decreased, with modifications in the expression of proteins linked to pulmonary vasodilation, consistent with low pulmonary vascular resistance. In summary, Cinaciguat, an activator of sGC, induces cardiopulmonary modifications in chronically hypoxic and pulmonary hypertensive newborn lambs. Therefore, Cinaciguat is a potential therapeutic tool for reducing pulmonary vascular remodeling and/or right ventricular hypertrophy in pulmonary arterial hypertension syndrome.

## Introduction

Newborns are especially vulnerable to neonatal pulmonary hypertension (NPHT) due to changes in pulmonary circulation that occur in the fetal-to-neonatal transition (Rudolph, 1979). The common denominator of this syndrome is sustained pulmonary vasoconstriction associated with vascular and right ventricle remodeling, resulting in increased pulmonary arterial resistance and hypertension (Abman, 1999; Herrera et al., 2007). When this occurs in chronically hypoxic and pulmonary hypertensive neonates, remodeling can compromise the whole pulmonary vessels (Kuhr et al., 2012).

One of the main pathways of vascular vasodilation involves endothelial nitric oxide synthase. (eNOS), which produces nitric oxide (NO). The latter activates soluble guanylyl cyclase (sGC), thus increasing cyclic GMP (cGMP) production. In turn, cGMP activates cyclic GMP–dependent protein kinase (PKG), that phosphorylates the myosin light chain phosphatase (MLCP), resulting in myosin light chain (MLC) dephosphorylation and smooth muscle vasodilatation (Gao and Raj, 2010).

This pathway is also involved in preventing pulmonary vascular remodeling by reducing the expression of transcription factors, such as HIF-1, and their actions on the lung, among other mechanisms (Fiedler et al., 2002; Prabhakar and Semenza, 2012). Soluble guanylyl cyclase (sGC) requires heme iron in its reduced state (Fe^2+^) to be active (Stasch et al., 2002; Stasch et al., 2011). Once sGC is activated, it converts guanosine triphosphate (GTP) to cyclic guanosine monophosphate (cGMP), which has a myriad of physiological functions (Gao and Raj, 2010). When sGC becomes oxidized or when the heme group is lost, the enzyme does not respond to NO, thereby losing its capacity to increase cGMP, preventing vasodilation, and increasing the remodeling of small pulmonary arteries (Stasch et al., 2002; Stasch et al., 2011).

Chronically hypoxic and pulmonary hypertensive neonatal sheep born in the Alto Andino have increased NO production and eNOS protein expression. Nevertheless, they have a notable decrease in sGC protein expression and function, reflected by the low cGMP concentration in the lung (Herrera et al., 2008; Ferrada et al., 2015). Due to abnormal soluble guanylyl cyclase (sGC) expression and function, this pathological milieu may contribute to pulmonary arterial hypertension produced by pulmonary arterial vasoconstriction and remodeling.

NO inhalation therapy is one of the treatments for human neonates with pulmonary arterial hypertension. However, up to 40% of neonates with persistent pulmonary arterial hypertension do not respond to inhaled NO (iNO) therapy (Walsh-Sukys et al., 2000), highlighting the need for new therapeutic options. One candidate drug is Cinaciguat or BAY-582667, which activates sGC when the enzyme is oxidized or has lost the heme group (Stasch et al., 2002; Stasch et al., 2011). This drug was used acutely in fetal lambs, increasing pulmonary blood flow and reducing pulmonary vascular resistance (Chester et al., 2009). Cinaciguat also induced pulmonary vasodilation when acutely infused in lambs with experimental pulmonary hypertension produced by ligation of the ductusarteriosus (Chester et al., 2011). Based on these findings, we hypothesized that Cinaciguat reduces pulmonary vascular resistance by decreasing pulmonary arterial vasoconstriction and remodeling by activating soluble guanylyl cyclase, which is already reduced in its expression and function.

To test this hypothesis, we used an integrative approach to determine the effects of Cinaciguat in high altitude pulmonary hypertensive newborn lambs studied at 3,600 m. We determined: 1) *in vivo* pulmonary and systemic arterial blood pressure and resistance, cardiac output and heart rate, during the daily acute infusion of Cinaciguat; 2) *in vivo* pulmonary and systemic arterial blood pressure and resistance, cardiac output, and heart rate for 7 days before the daily acute infusion of Cinaciguat or vehicle and during a superimposed episode of acute hypoxia; 3) changes in the heart ventricle weight; histomorphology of pulmonary arteries (vascular density, histology, immunohistochemistry, and muscle layer cellular density); 4) expression of molecules related to cellular division, apoptosis (heart and lung) and vasodilator function (lung) and 5) *ex vivo* vasoreactivity response to KCl and vasodilators in isolated small pulmonary arteries.

## Materials and Methods

The Ethics Committee of the Faculty of Medicine, University of Chile, approved all the experimental protocols (CBA #0643 FMUCH). Animal care, maintenance, procedures, and experimentation were performed following the Guide for the Care and Use of Laboratory Animals published by the US National Institutes of Health (NIH Publication No. 85-23, revised 1996) and American Physiological Society’s Guiding Principles in the Care and Use of Animals.

### Animals

Twelve highland newborn lambs, whose ancestors lived for several generations at high altitude, were studied at the Putre Research Station (3,600 m above sea level), International Center for Andean Studies (INCAS), University of Chile, in the Region of Arica and Parinacota, Chile. The lambs were conceived, gestated, and born at Putre and were kept with their dams at the Station´s animal facilities. At birth, six lambs (three females and three males) were randomly allocated to the Control group (receiving vehicle) and six lambs (four females and two males) to the Cinaciguat-treated (BAY 58-2667, LC Laboratories, MA, United States) group. The lambs were instrumented at 3 days old, and from 4 to 10 days old, they were given Cinaciguat (35 μg kg^−1^day^−1^, according to Chester et al., 2011) or vehicle [dimethyl sulfoxide (DMSO): 0.9% NaCl, 1:10] administered into the pulmonary artery daily. The day after the last treatment (day 11), lambs were subjected to a superimposed episode of acute hypoxia. The following day (day 12), lambs were euthanized with sodium thiopentone 100 mg kg^−1^ i.v. (Tiopental; Laboratorio Biosano, Santiago, Chile) and tissues were collected. Body weights at euthanasia were 5.68 ± 0.36 kg and 5.90 ± 0.25 kg, Control and Cinaciguat groups, respectively. Afterwards, the ewes were returned to the flock.

### Surgical Preparation

All surgical procedures were performed under aseptic conditions. At 3 days old, lambs were instrumented under general anesthesia with ketamine, 10 mg kg^−1^ i.m. (Ketostop; Drag Pharma-Invectec, Santiago, Chile), xylazine, 0.1–0.5 mg kg^−1^ i.m. (Xilazina 2%, Laboratorio Centrovet, Santiago, Chile), and atropine (0.04 mg kg^−1^ i.m. (Atropina Sulfato; Laboratorio Chile, Santiago, Chile), with additional local infiltration of 2% lidocaine (Dimecaina; Laboratorio Beta, Santiago, Chile). Polyvinyl catheters (1.2 mm internal diameter) were placed into the descending aorta and inferior vena cava. A Swan-Ganz catheter (Swan-Ganz 5 French, Edwards Lifesciences LLC, Irvine, CA, United States) was placed in the pulmonary artery. All catheters were filled with heparin solution (1000 IU ml^−1^ in 0.9% NaCl), exteriorized and kept in a cloth pouch sewn onto the skin. Oxytetracycline, 20 mg kg^−1^, i.m. (Liquamicina LA, Pfizer, Chile), and sodium metamizole 0.1 mg kg^−1^, s.c. (Metamizol sódico, Laboratorio Chile, Chile) were given immediately after surgery and during the next 3 postoperative days.

### Daily Recordings of Cardiopulmonary Variables During Cinaciguat Treatment Under High Altitude Chronic Hypoxia

Starting at day 4 (1 day after surgery) and for the following 7 days, lambs were brought to the Station laboratory every morning. All *in vivo* measurements were performed in unanesthetized neonatal lambs comfortably placed in a home-made canvas sling. Catheters were attached to pressure transducers and to a data acquisition system connected to a computer (Powerlab/8SP System and Chart v4.1.2 Software; ADInstruments, NSW, Australia) to continuously recording pulmonary and systemic pressures and heart rate. After 15 min of basal recordings, lambs were given a bolus of Cinaciguat or vehicle for 3 min and recordings were continued during the next hour. Blood gases: arterial pH (pHa), arterial oxygen partial pressure (PaO_2_), arterial CO_2_ partial pressure (PaCO_2_), hemoglobin saturation (%Sat Hb), hemoglobin concentration (Hb) (IL-Synthesis 25, Instrumentation Laboratories, Lexington, MA, United States) were measured at fixed intervals during the protocol, and values were corrected to 39°C. Heart rate, mean pulmonary and systemic arterial pressures (HR, mPAP & mSAP, respectively) were obtained from the records. We calculated pulmonary (PVR) and systemic vascular resistance (SVR) as described previously (Herrera et al., 2007). Additionally, every 15 min we measured the cardiac output (CO) by thermodilution, injecting 3 ml of chilled (4°C) 0.9% NaCl into the pulmonary artery via the Swan-Ganz catheter connected to a cardiac output computer (COM-2 model; Baxter, Edwards Critical-Care Division, Irvine, CA, United States). Lambs were returned to their mothers after each session.

### Cardiopulmonary Response to Superimposed Acute Hypoxia

At 11 days of age, 24 h after the last treatment, the newborn lambs were connected as described above and subjected to a superimposed episode of acute hypoxia, consisting of 30 min of breathing room air, 30 min of hypoxia (PaO_2_ ∼ 30 ± 2 mmHg), followed by 30 min under room air (recovery). Hypoxia was induced by passing approximately 20 L min^−1^ of 10% O_2_ and 2%–3% CO_2_ in N_2_ through a loosely tied transparent polyethylene bag placed over the animal’s head. Blood gases, hemoglobin saturation, and hemoglobin concentration were measured at 15 min intervals during the protocol. Mean PAP, mSAP, and HR were measured continuously, and PVR and SVR were calculated (Herrera et al., 2007). Additionally, cardiac output (CO) was measured at 15 min intervals. After recovery, lambs were returned to their mothers.

### Euthanasia and Organ Dissection

At 12 days of age, after the episode of acute hypoxia and 48 h after the last Cinaciguat treatment or vehicle, the lambs were euthanized with an overdose of sodium thiopentone 100 mg kg^−1^ i.v. (Tiopental; Laboratorio Biosano, Santiago, Chile). Lungs and hearts were dissected, weighed, and immediately immersed in cold 0.9% NaCl. The right and left lungs were separated. The right lung was processed for wire myographic studies and molecular biology, and the left lung for histology, as described below. The heart was dissected; right and left atriums, right and left ventricles, and septum, were weighed. Right ventricular hypertrophy index (RVHI; [right ventricle weight/left ventricle weight + septum weight] × 100) and percentages of right and left ventricle and septum vs. newborn weight were calculated. Pieces of the left lung and right ventricle were stored in liquid nitrogen for molecular biology measurements. All other organs were dissected and weighed.

### Wire Myography

Procedures were performed at Putre Research Station. Small pulmonary arteries between 150–400 µm were dissected from the caudal lobe of the right lung under a magnifying glass (Nikon 102, ×4). Isolated arteries were mounted in a wire myograph (DMT 610M, Denmark), maintained at 37°C in Krebs buffer aerated with 95% O_2_—5% CO_2_ (Herrera et al., 2007). The optimal diameter was obtained by stretching the vessel in a stepwise manner to a standardized tension equivalent to a physiological transmural pressure of 25 mmHg (Herrera et al., 2010). A concentration-response curve (CRC) was constructed for KCl. This CRC was analyzed in terms of maximal contraction and sensitivity. The contractile response was measured in terms of tension (N/m) using the Boltzmann equation. Sensitivity was calculated as EC50, the concentration at which 50% of the maximal response was obtained (Herrera et al., 2008). CRCs were constructed for SNP, sildenafil, and NS1619. These CRCs were analyzed in terms of maximal relaxation and sensitivity. Vasodilatation responses were determined in terms of maximal relaxation (%) by fitting experimental data to a sigmoidal equation (Ebensperger, 2011). Relaxation curves were performed after adding serotonin 10^−6^ M as a constrictor agent, and the responses were expressed as a percentage relative to the maximal contraction induced by serotonin (Ebensperger, 2011). Sensitivity was calculated as pD2, where pD2 = −log[EC50] (Neubig et al., 2003). In a few cases arteries were not suitable for the experiments, thus SNP experiments were performed in 5 Control and 5 Cinaciguat lambs, sildenafil experiments in 6 Control and 5 Cinaciguat lambs, and NS1619 in 5 Control and 6 Cinaciguat lambs.

### Pulmonary Histomorphometry Measurements

The left lung was isolated, excised, and perfused with a cold 0.9% NaCl solution. Lung slices were cut from the central part of the lobe. About 1 cm^3^ pulmonary blocks were fixed in 4% paraformaldehyde in PBS for 24 h at room temperature, embedded in paraffin, cut into 5 µm sections and stained with van Gieson. We measured lung artery density, percentages of vascular smooth muscle and adventitia areas, and muscle layer cellular density. To determine lung artery density, we counted arteries with an internal diameter between 12 and 1,000 µm in the parenchymal lung area, excluding the bronchial and cartilaginous areas. The total area of the analyzed histological sections was similar between Cinaciguat and Control groups. Slides were digitized using the NanoZoomer XR scanner (Hamamatsu Photonics, Hamamatsu, Japan). The visualization and determination of the region of interest (ROIs) for counting arteries and measuring pulmonary parenchyma areas was performed by the QuPath v0.3.0 program (Bankhead et al., 2017). Vascular density was measured in the whole slide by counting the number of arteries and dividing it by the parenchyma surface in mm^2^. The percentages of vascular smooth muscle and adventitia areas were calculated (Minamino et al., 2001; Torres et al., 2015; Castillo-Galán et al., 2016; Lopez et al., 2016; Astorga et al., 2018). Six arteries (100–150 µm of inner diameter) per lung were analyzed, and an average of five measurements from each artery was recorded. Images of parenchymal arterioles were acquired using a workstation (Olympus trinocular microscope-BX51 plus digital camera QimaginG O3) linked to ImagePro software 6.3, and the vascular areas were calculated using the same software. Smooth muscle area (%) = [(external muscle area − internal area)/external muscle area] × 100, where the external muscle area and the internal area are the outer and inner limits of the tunica media, respectively. Adventitial area (%) = [(external adventitial area − external muscle area)/(external adventitial area)] × 100, where the external adventitial area and external muscle area are the outer boundary of the adventitia and the outer boundary of the tunica media, respectively. We counted nuclei in the smooth muscle area to visually calculate cellular density. The average count of each small pulmonary artery was divided by the previously measured total smooth muscle area (cells/μm^2^) × 100.

### Ki67 Immunohistochemistry

The proliferation of smooth muscle cells in small pulmonary arteries (100–150 µm internal diameter) was measured as described by Castillo-Galán et al., 2016. Briefly, immunohistochemical detection for Ki67 was performed with commercial antibodies (Clone MIB-1, Dako, Glostrup, Denmark, dilution 1:100). Antigen retrieval was performed at 100°C with citrate buffer pH: 6, 1X (Dako). Primary antibody incubations were performed overnight at 4°C and visualized using an HRP-diaminobenzidine kit (Envision TM+; Dako). We considered cells Ki67 positive when they had a brown nucleus and Ki67 negative cells as those with a blue nucleus. We visually identified Ki67 positive arteries in 4 Control and 4 Cinaciguat newborns. In these arteries we calculated the percentage of Ki67 positive cells in the medial layer of small pulmonary arteries as the ratio of positive Ki67 nuclei versus total nuclei count (Castillo-Galán et al., 2016).

### Western Blot

Lung and right ventricle lysates were prepared from frozen tissue and resolved by electrophoresis in SDS-polyacrylamide gels and electrotransferred to a nitrocellulose membrane (Lopez et al., 2016). After nonspecific binding blocking by incubating for at least 1 h with 4% skim-milk in phosphate-buffered saline (PBS) membranes were incubated with the following antibodies: anti-p21, (Cat. No. sc-397, polyclonal; Santa Cruz Biotechnology); anti-eNOS (Cat. No. 610296, monoclonal; BD Biosciences); anti-HO-1 (Cat. No. 10R-H112A, monoclonal; Fitzgerald); anti-sGC-α1 (Cat. No. sc-37502, monoclonal, Santa Cruz Biotechnology); anti-sGC-β1 (Cat. No. sc-514183, monoclonal; Santa Cruz Biotechnology); anti-PKG-1α (Cat. No. ADI-KAP-PK005, polyclonal; Enzo Life Sciences); anti-MLC20 (Cat. No. 3672S; Cell Signaling Technology); anti-PDE5 (Cat. No. 611498, monoclonal; BD transduction Laboratories); anti-BK_Ca_ (Cat. No. AB_10698180 clone L6/60, monoclonal; Neuromab, Davis, CA, United States); and anti-β-Actin (Cat. No. MA1-91399, monoclonal; Thermo Fisher Scientific). In addition, lung and right ventricle lysates, were incubated with anti-Phospho-p38 MAPK (Cat. No. 9215, polyclonal; Cell Signaling); anti-p38 MAPK (Cat. No. 9212, polyclonal; Cell Signaling); anti-Phospho p53 (Cat. No. sc-56173, monoclonal; Santa Cruz Biotechnology); anti-p53 (Cat. No. sc-6243, monoclonal; Santa Cruz Biotechnology); anti-Cyclin D1 (Cat. No. sc-717, polyclonal; Santa Cruz Biotechnology); anti-PARP (Cat. No. 556362, monoclonal; BD Pharmingen) and anti-β-Actin (Cat. No. MA1-91399, monoclonal; Thermo Fisher Scientific). Bands were revealed by incubating with HRP-conjugated anti-rabbit IgG or anti-mouse IgG secondary antibodies (both from Thermo Fisher Scientific, Rockford, IL, United States; used at a 1:5,000 dilution). Signals were detected with an enhanced chemiluminescent (ECL) (SuperSignal West Pico Chemiluminescent Substrate; Thermo Fisher scientific, Rockford, IL, United States) and autoradiography, and quantified by densitometric analysis using the Scion Image Software (Scion Image Beta 4.02 Win; Scion Corporation, MD, United States). The relative amount of proteins was calculated as the intensity ratio of the protein band of interest vs. the intensity of the β-Actin band. All these antibodies were used in previous studies in sheep and llama: anti-eNOS and anti-β-Actin (Torres et al., 2015); anti-p21, anti-Cyclin D1, anti-PDE5, anti-BKCa, anti-PKG-1α and anti-MLC20 (Herrera et al., 2019); anti-p53 (Yang et al., 2012); anti-PARP (Ebensperger et al., 2005). Western blot conditions for each antibody are indicated in Table 1. All immunoblot analyses were performed with the same tissue homogenates. Therefore, the β-Actin image corresponding to these preparations was re-used for illustrative purposes.

**TABLE 1:** Westernblot conditions for each antibody.

Antibody	Weight (kDa)	Protein loaded (µg)	Poli-acrylamide gel (%)	Antibody dilution	Incubation time
anti-p21	21	50 (L)	10	1:750	Overnight
anti-eNOS	140	10 (L)	10	1:1,000	60 min.
anti-HO-1	32	10 (L)	8	1:1,000	Overnight
anti-sGC-α1	72	10 (L)	10	1:5,000	Overnight
anti-sGC-β1	65	10 (L)	10	1:5,000	Overnight
anti-PKG-1α	75	5 (L)	10	1:1,000	60 min.
anti-MLC20	18	20 (L)	14	1:1,000	Overnight
anti-PDE5	95	10 (L)	10	1:1,000	60 min.
anti-BKCa	130	20 (L)	10	1:1,000	120 min.
anti-β-Actin	43	0.5 (L)	10	1:5,000	60 min.
anti-β-Actin	43	20 (RV)	10	1:5,000	60 min.
anti-Cyclin D1	37	20 (L and RV)	14	1:750	Overnight
anti-Phospho p38 MAPK	43	60 (L and RV)	10	1:200	Overnight
anti-p38 MAPK	43	10 (L and RV)	14	1:3,000	90 min.
anti-Phospho p53	55	50 (L and RV)	10	1:300	120 min.
anti-p53	55	20 (L and RV)	10	1:300	120 min.
anti-PARP	116, 89	50 (L and RV)	10	1:500	120 min.

L, lung; RV, right ventricle. Overnight incubation, approximately 18 h at 4°C.

### Real-Time PCR

Bax and Bcl2 messenger RNA was measured by qRT-PCR in lung tissue homogenates. Total RNA was obtained using the SV Total RNA Isolation kit (Promega, Madison, WI, United States). cDNA was synthesized by M-MLV Reverse Transcriptase (200 U/μl) (Catalog No. 28025013, Invitrogen, Carlsbad, CA, United States) as described in Seron-Ferre et al. (2017). The primers used for Bax were: forward 5′- CCG​ACG​GCA​ACT​TCA​ACT​GG -3′ and reverse 5′- GAT​CAA​CTC​GGG​CAC​CTT​GG -3′. Primers for Bcl2 were forward 5′-GGA​TGA​CCG​AGT​ACC​TGA​AC -3′ and reverse 5′- GGC​CAT​ACA​GCT​CCA​CAA​AG -3'. Quantitative real-time PCR assays were performed in a StepOne thermal cycler (Applied Biosystems, CA, United States). The reaction procedure was as follows: initial denaturation step at 95°C for 15 min and 40 cycles (95°C for 30 s; 60°C for 30 s; 72°C for 30 s). Sample quantification was performed using a standard curve constructed with serial dilutions of known quantities of PCR products for each gene. The results were expressed as the Bax/Bcl2 ratio.

### Measuring cGMP Concentration in Lung Tissue

Frozen lung tissue was homogenized, total protein was extracted, and cGMP was measured following the manufacturer´s instructions (Cayman Chemical, Cyclic GMP ELISA Kit Item N° 581021). Results were expressed in pmol/mg proteins. Protein content was determined using the Bradford method (Bradford, 1976) with bovine serum albumin as the standard.

### Statistical Analysis

Data were expressed as means ± SEM. *In vivo* groups were compared using the one-way ANOVA followed by the post hoc Dunnett/Newman-Keuls test, area under the curve with a non-parametric Mann Whitney test, or two-way ANOVA followed by the post hoc Newman-Keuls test, as appropriate. *Ex vivo* and *in vitro* studies were analyzed using the non-parametric Mann Whitney test. Analyses were performed using Prism 6.01, GraphPad Software, La Jolla, CA. Differences were considered statistically significant when *p* ≤ 0.05 (Glantz and Slinker, 2001).

## Results

### The Response of Cardiovascular Variables to Cinaciguat Infusion

**FIGURE 1:**
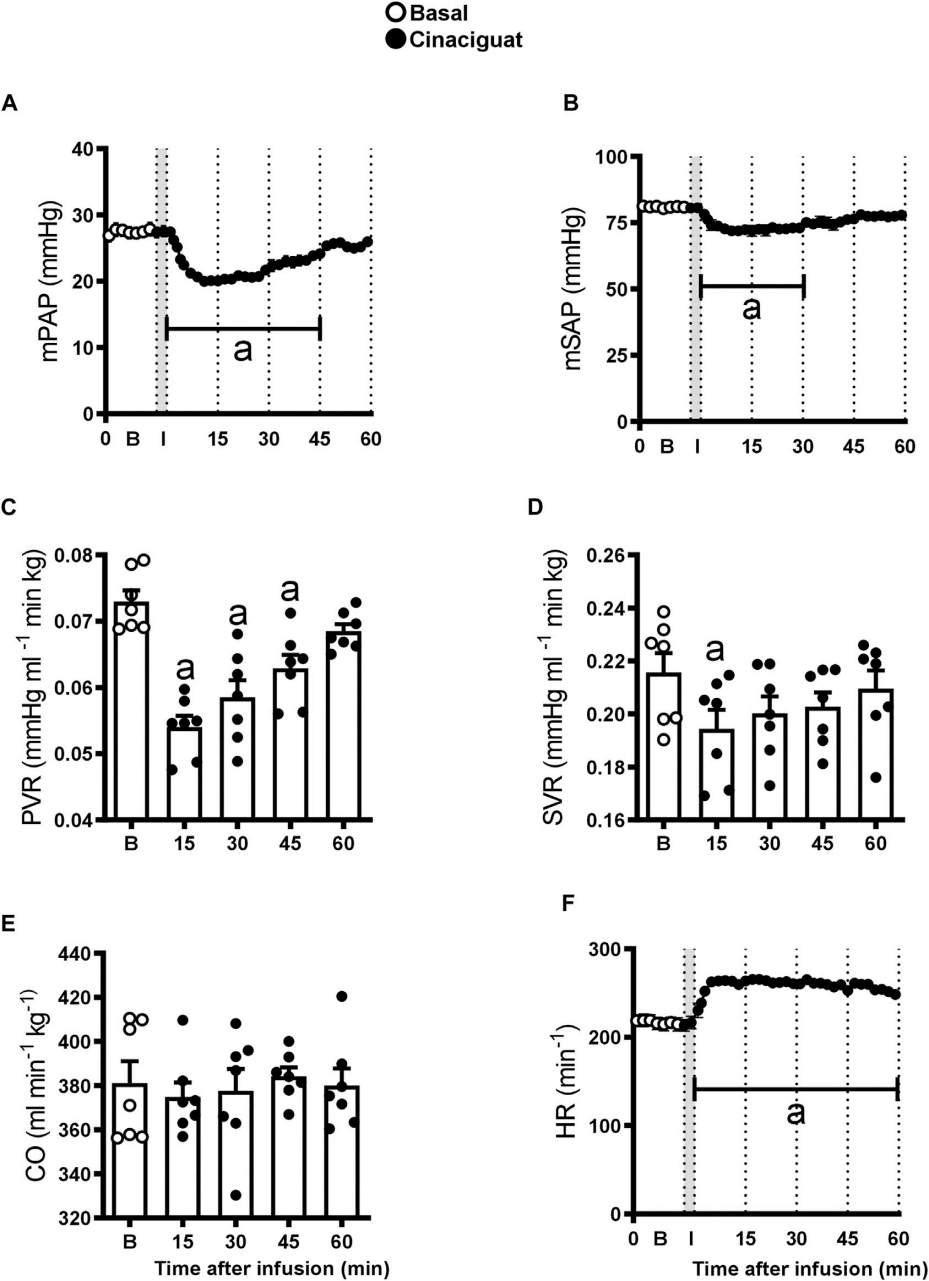
Acute effects of a 3 min infusion of Cinaciguat on the cardiopulmonary and systemic circulations of 6 chronically hypoxic and pulmonary hypertensive neonatal lambs. Infusions were performed in 7 successive days and results are means ± SEM of each variable at indicated times. Open circles: Basal values **(B)**; Black circles, values after Cinaciguat infusion. Mean pulmonary arterial pressure [(mPAP, **(A)**], mean systemic arterial pressure [mSAP, **(B)**]; pulmonary vascular resistance [PVR, **(C)**]; systemic vascular resistance [SVR, **(D)**]; cardiac output [CO, **(E)**], and heart rate [HR, **(F)**] were recorded for 60 min. In the *X* axis, B, basal; I, infusion. Significant differences (*p* ≤ 0.05): a vs. basal period.

Newborn lamb cardiovascular variables responded to Cinaciguat bolus, but the duration of the response differed between the variables (Figure 1). Mean PAP and PVR decreased by approximately 27% compared to their basal values in the first 10 min after Cinaciguat administration, returning to basal levels at 45 min (*p* ≤ 0.05, Figures 1A,C, respectively). The response of mSAP and SVR consisted of an initial 10% decrease that lasted 30 and 15 min, respectively (*p* ≤ 0.05, Figures 1B,D). In contrast, CO was maintained during Cinaciguat treatment (*p* < 0.05, Figure 1E), whereas HR increased by 22% and lasted for 60 min post Cinaciguat infusion (*p* ≤ 0.05, Figure 1F). Arterial pH and blood gases did not vary with the acute bolus injection of Cinaciguat. Also, cardiorespiratory variables, arterial pH, and blood gases did not change in response to vehicle administration (data not shown).

**FIGURE 2:**
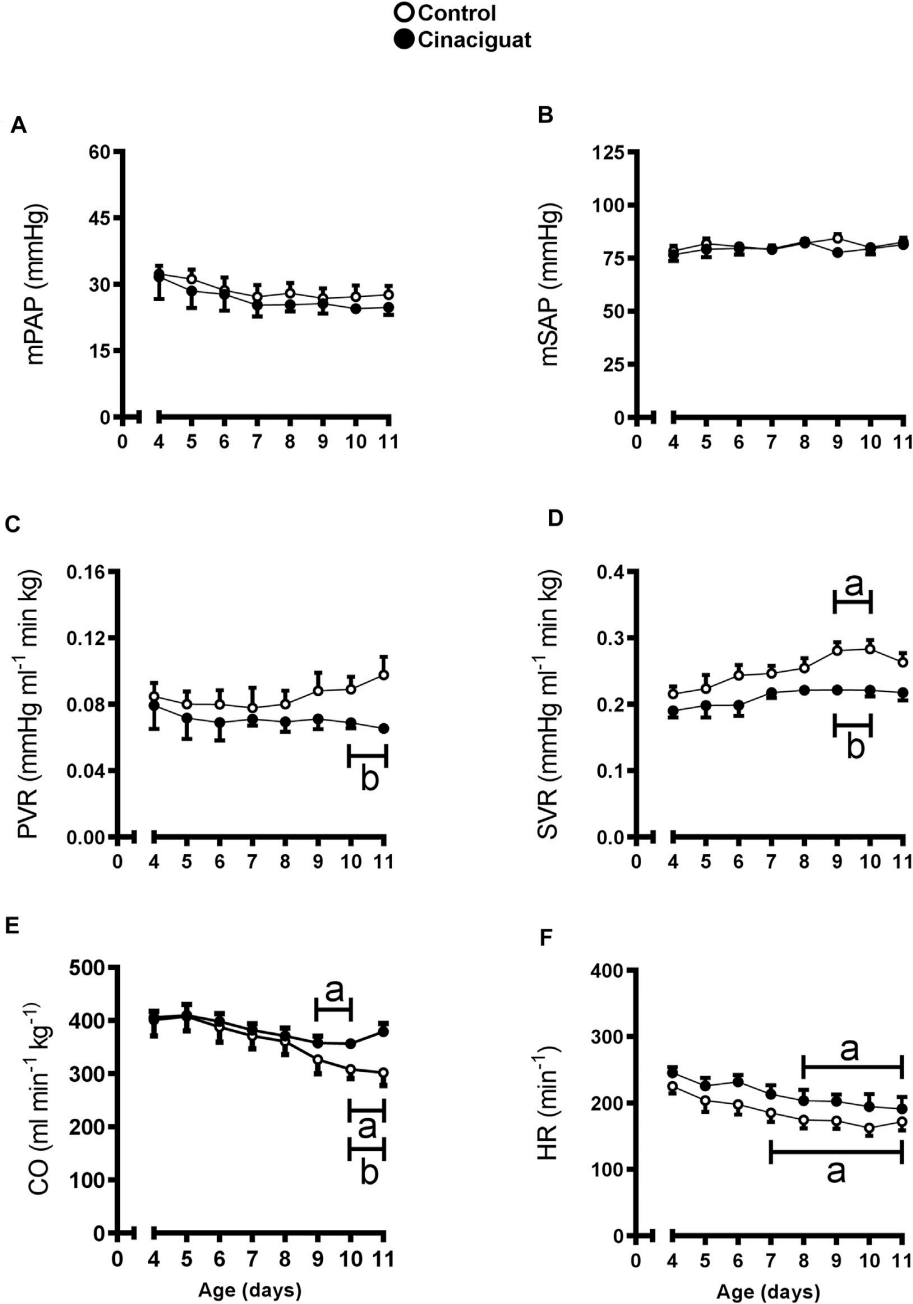
Time-course of basal cardiopulmonary and systemic circulation variables in chronically hypoxic and pulmonary hypertensive neonatal lambs treated with a daily bolus of Cinaciguat. Mean pulmonary arterial pressure [mPAP, **(A)**]; mean systemic arterial pressure [mSAP, **(B)**]; pulmonary vascular resistance [PVR, **(C)**]; systemic vascular resistance [SVR, **(D)**]; cardiac output [CO, **(E)**], and heart rate [HR, **(F)**]. Control (Open circles, *n* = 6) and Cinaciguat-treated lambs (Black circles, *n* = 6). Values are means ± SEM. Significant differences (*p* ≤ 0.05): a vs. basal period, b vs. Control group.

Daily values of cardiovascular variables were registered before Cinaciguat or vehicle administration from 4 to 10 days of age (Figure 2). At 10 and 11 days of age, basal PVR was lower in Cinaciguat than in Control newborns (*p* ≤ 0.05, Figure 2C), nevertheless, basal mPAP was similar to Controls from 4 to 11 days (Figure 2A). This decrease in PVR coincided with CO maintenance, although it also decreased in the Controls (*p* ≤ 0.05, Figure 2E). Mean SAP and HR were similar between groups (Figures 2B,F, respectively), and SVR was lower in Cinaciguat treated lambs at 9 and 10 days old (Figure 2D).

**FIGURE 3:**
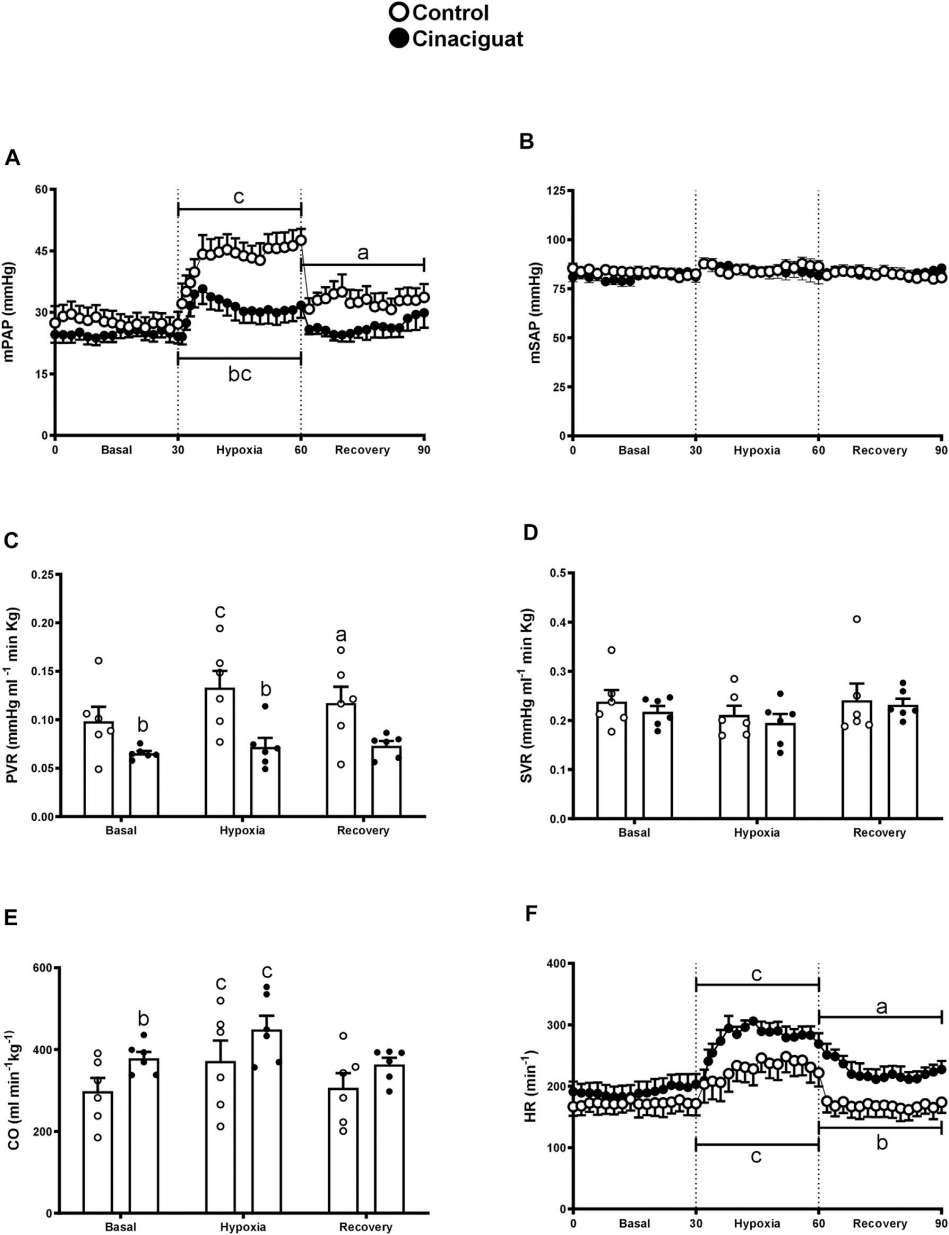
Effect of Cinaciguat treatment on cardiopulmonary and systemic circulation following a superimposed episode of acute hypoxia in chronically hypoxic and pulmonary hypertensive neonatal lambs. Mean pulmonary arterial pressure [mPAP, **(A)**], mean systemic arterial pressure [mSAP, **(B)**], pulmonary vascular resistance [PVR, **(C)**], systemic vascular resistance [SVR, **(D)**], cardiac output [CO, **(E)**], and heart rate [HR, **(F)**]. Control (Open circles, *n* = 6) and Cinaciguat-treated lambs (Black circles, *n* = 6). Values are means ± SEM. Significant differences (*p* ≤ 0.05): a vs. basal period, b vs. Control group; c, hypoxia vs. basal and recovery periods.

The difference between Cinaciguat and Control newborns was amplified at day 11, 24 h after the last dose of Cinaciguat when the newborns were subjected to a superimposed episode of acute hypoxia (Figure 3). Hypoxia increased mPAP in both groups; however, the response in the Cinaciguat group was markedly lower than the Control group (*p* ≤ 0.05, Figure 3A). Cinaciguat treated newborns had a lower basal PVR and higher CO on the day before the last treatment. Pulmonary vascular resistance did not increase during the hypoxic episode in the Cinaciguat group, unlike Control newborns. Also, the Cinaciguat lambs still maintained a low PVR during recovery (Figure 3C). Cardiac output and HR increased in both groups during hypoxia (*p* ≤ 0.05, Figures 3E,F, respectively). Nonetheless, HR was higher during the recovery time in the Cinaciguat group than in the Controls (*p* ≤ 0.05, Figure 3F). The effects of hypoxia in pulmonary circulation were different from those found in systemic circulation. There were no changes in mSAP and SVR during basal, hypoxia, and recovery periods in both groups (Figures 3B,D, respectively). The blood gases and pHa of Control lambs were similar to those reported in previous studies (Lopez et al., 2016). However, [Hb] was higher during the recovery period in the Cinaciguat group compared to Controls (12.47 ± 0.31 vs. 10.44 ± 0.78, respectively, *p* ≤ 0.05, Table 2).

**TABLE 2:** Aortic pH and arterial blood gases during a superimposed episode of acute hypoxia in chronically hypoxic and pulmonary hypertensive neonatal lambs.

Variables	Treatment	Basal	Hypoxia	Recovery
PHa	Control	7.49 ± 0.01	7.45 ± 0.01	7.44 ± 0.02
	Cinaciguat	7.48 ± 0.01	7.47 ± 0.03	7.45 ± 0.02
PaO_2_ (mmHg)	Control	43.02 ± 0.75	29.80 ± 0.27c	46.90 ± 1.57
	Cinaciguat	42.36 ± 1.66	29.58 ± 0.83c	44.34 ± 3.02
PaCO_2_ (mmHg)	Control	28.96 ± 1.00	28.09 ± 1.21	26.51 ± 1.27
	Cinaciguat	30.70 ± 0.80	30.50 ± 1.00	29.20 ± 1.10
Sat Hb (%)	Control	73.17 ± 0.96	47.62 ± 1.99c	75.72 ± 1.17
	Cinaciguat	75.59 ± 2.67	43.08 ± 3.28c	78.54 ± 3.05
[Hb] (g dl^−1^)	Control	10.79 ± 0.78	10.87 ± 0.82	10.44 ± 0.78
	Cinaciguat	11.83 ± 0.56	12.58 ± 0.48a	12.47 ± 0.31ab

Arterial pH, pHa; arterial oxygen partial pressure, PaO_2_ (mmHg); arterial CO_2_ partial pressure, PaCO_2_ (mmHg); saturation of hemoglobin with oxygen, Sat Hb (%); and hemoglobin concentration [Hb] (g dl^−1^). Control (*n* = 6) and Cinaciguat-treated newborn lambs (*n* = 6). Values are means ± SEM. Significant differences (p ≤ 0.05): a vs. basal period, b vs. Control group, c, hypoxia vs. basal and recovery periods.

### The Effects of Cinaciguat on the Heart and Other Organs

**FIGURE 4:**
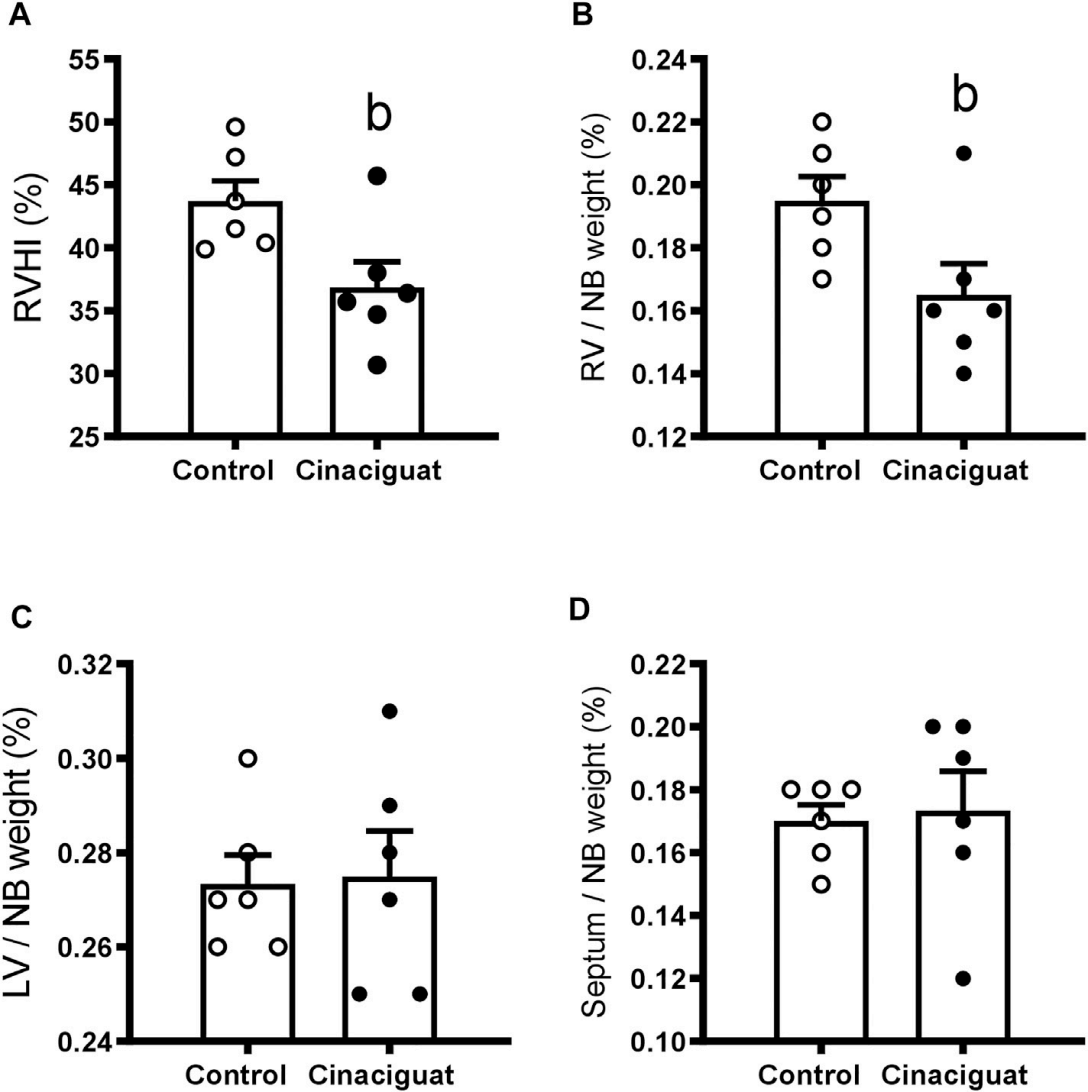
Effect of Cinaciguat treatment on heart ventricles of chronically hypoxic and pulmonary hypertensive neonatal lambs. Percentages of right ventricular hypertrophy index [(right ventricle/(left ventricle + septum) ×100] **(A)**; [(right ventricle weight/newborn weight) x 100] **(B)**; [(left ventricle weight/newborn weight) x 100] **(C)**, and [(septum weight/newborn weight) ×100] **(D)**. Control (Open circles, *n* = 6) and Cinaciguat-treated lambs (Black circles, *n* = 6). Values are means ± SEM. Significant difference (*p* ≤ 0.05): b vs. Control group.

Organs were collected the day after the superimposed episode of acute hypoxia. Cinaciguat treatment decreased the percentage of right ventricle/newborn weight in relation to Controls (0.17% ± 0.01% vs*.* 0.20% ± 0.01%, respectively, *p* < 0.05, Figure 4B), without changing the percentages of the left ventricle and septum/newborn weight (Figures 4C,D, respectively), resulting in a lower right ventricular hypertrophy index (Cinaciguat 36.87% ± 2.03% vs. Control 43.72% ± 1.60%, *p* ≤ 0.05, Figure 4A). The right and left atrium showed no differences in absolute weight nor in relation to body weight between the two groups (data not shown). Cinaciguat treated newborns presented an increased percentage of the kidney weight/body weight compared to Control newborns (Cinaciguat 0.79% ± 0.04% vs. Control 0.65% ± 0.03%, *p* ≤ 0.05). To measure changes in right ventricle cell proliferation and apoptosis, we examined the expression of some proteins involved in cell division, such as Phospho-p38 MAPK/p38 MAPK, Phospho-p53/p53, Cyclin D1/β-Actin, and the apoptosis marker Poly ADP-ribose polymerase (PARP) for the proteolytic fragment (P116-P89). There were no significant changes in the levels of these proteins (Supplementary Figure S1; Supplementary Table S1) nor in the weight of other organs (data not shown) between Cinaciguat and Control lambs.

Altogether, these data showed that Cinaciguat had important effects *in vivo* on pulmonary circulation, decreasing the responses to superimposed hypoxia. In addition, Cinaciguat decreased right ventricle size, which seems unrelated to cell proliferation and apoptosis. The possible effects of Cinaciguat in the kidneys remain to be explored.

### The Effects of Cinaciguat Administration on Pulmonary Arteries

The effects of Cinaciguat on small pulmonary arteries were evaluated at the histometric and functional levels. In addition, proteins involved in the NO-sGC-cGMP pathway were evaluated in the lung.

**FIGURE 5:**
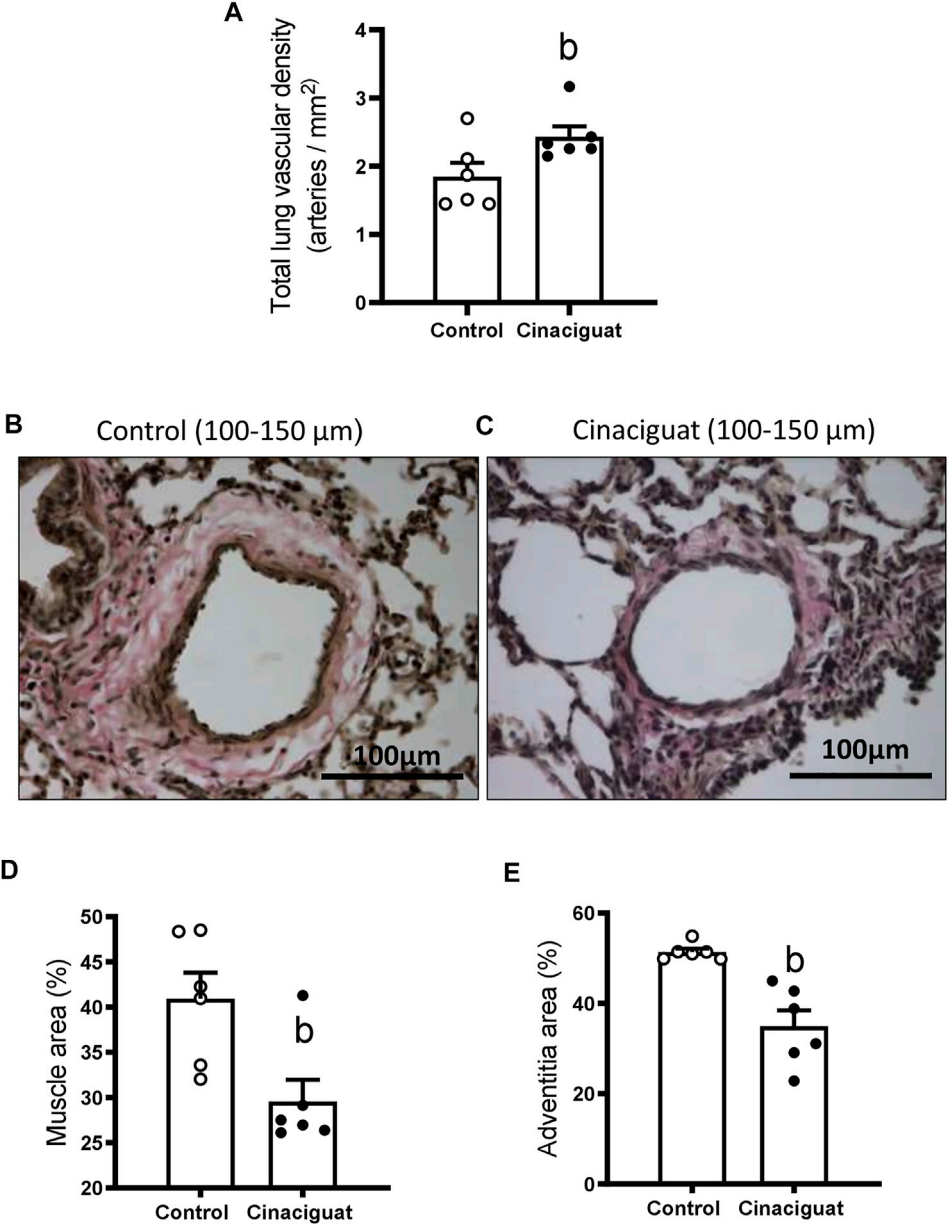
Effect of Cinaciguat treatment on pulmonary vascular density and small pulmonary artery remodeling in chronically hypoxic and pulmonary hypertensive neonatal lambs. Total lung vascular density (arteries/mm^2^) **(A)**. Representative micrograph of small pulmonary arteries, between 100 and 150 μm of luminal diameter, from vehicle group **(B)** and + Cinaciguat group **(C)**, van Gieson staining. Brown area: muscular layer; pink area surrounding muscle: adventitia layer. Bar: 100 μm. Magnification: × 40. Percentage of muscle area **(D)**, percentage of adventitia area **(E)**. Control (Open circles, *n* = 6) and Cinaciguat-treated lambs (Black circles, *n* = 6). Values are means ± SEM. Significant difference (*p* ≤ 0.05): b vs Control group.

The lambs treated with Cinaciguat showed higher vascular density (numbers of arteries per parenchyma surface) compared to the Control group (2.43 ± 0.15 arteries/mm^2^ vs. 1.84 ± 0.20 arteries/mm^2^, respectively, *p* ≤ 0.05, Figure 5A). In addition, there were changes in the structure of small pulmonary arteries (SPA) between 100–150 μm of internal diameter. The percentage of muscle and adventitia area decreased in the Cinaciguat group compared to the Control group (Figures 5B,C, respectively). The muscle area was 25.15 ± 1.40% in the Cinaciguat treated lambs vs. 40.01 ± 4.20% in the Controls (*p* ≤ 0.05, Figure 5D). The adventitia area was 32.25 ± 3.98% in Cinaciguat lambs compared to 48.62% ± 1.92% in Controls (*p* ≤ 0.05, Figure 5E).

**FIGURE 6:**
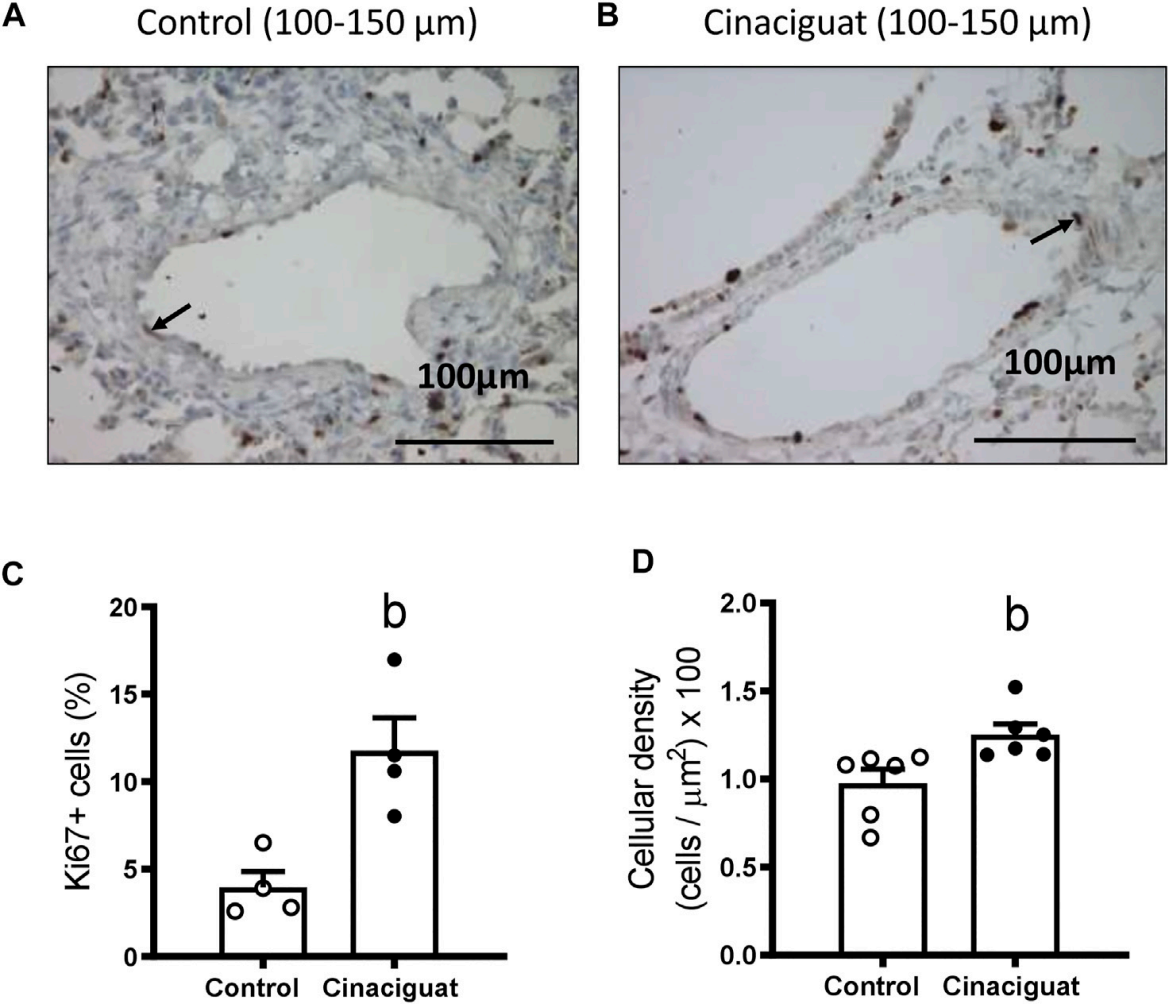
Effect of Cinaciguat treatment on muscle layer proliferation (Ki67 expression) and cellular density in small pulmonary arteries of chronically hypoxic and pulmonary hypertensive neonatal lambs. Representative micrograph of small pulmonary arteries, between 100 and 150 μm of luminal diameter, from Control **(A)** and Cinaciguat-treated lambs **(B)**. Ki67+ cells are shown with an arrow. Bar: 100 μm. Magnification: ×40. Percentage of Ki67+ cells of small pulmonary arteries **(C)**. Control (Open circles, *n* = 4) and Cinaciguat-treated lambs (Black circles, *n* = 4). Values are means ± SEM. Significant difference (*p* ≤ 0.05): b vs. Control group. Cellular density in muscle layer (cells/µm^2^) ×100 **(D)**. Control (Open circles, *n* = 6) and Cinaciguat-treated lambs (Black circles, *n* = 6). Values are means ± SEM. Significant difference (*p* ≤ 0.05): b vs. Control group.

**FIGURE 7:**
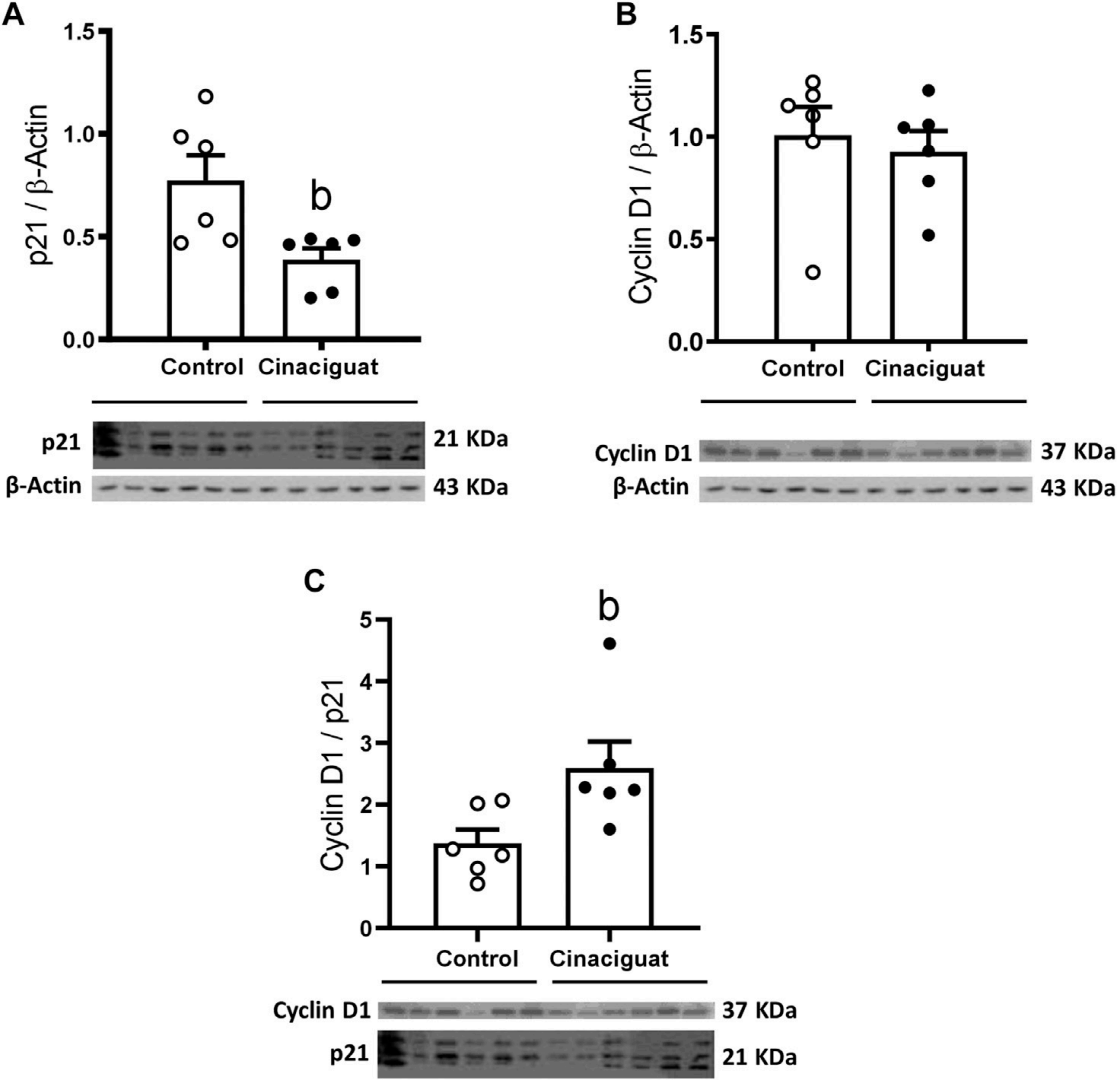
Effect of Cinaciguat treatment on pulmonary protein expression of cell proliferation markers in chronically hypoxic and pulmonary hypertensive neonatal lambs. WB of p21/β-Actin **(A)**, Cyclin D1/β-Actin **(B)**, and Cyclin D1/p21 ratio **(C)**. The β-Actin image was re-used for illustrative purposes. Control (Open circles, *n* = 6) and Cinaciguat-treated lambs (Black circles, *n* = 6). Values are means ± SEM. Significant difference (*p* ≤ 0.05): b vs. Control group.

We measured Ki67 positive cells in the muscle area of small pulmonary arteries between 100 and 150 μm of internal diameter to assess if these modifications involved changes in proliferation (Figures 6A,B). The Cinaciguat group had a higher % of Ki67 positive cells than Controls (11.78 ± 1.88% vs. 3.96 ± 0.90%, respectively, *p* ≤ 0.05, Figure 6C). Consistent with the previous result, the percentage of muscle layer cellular density increased in the Cinaciguat group compared to the Control group (1.25 ± 0.07% vs. 0.98 ± 0.08%, respectively, *p* ≤ 0.05, Figure 6D). Further, p21/β-Actin protein expression was lower in the Cinaciguat groups compared to the Controls (0.39 ± 0.06 vs. 0.77 ± 0.12, respectively, *p* ≤ 0.05, Figure 7A). Cyclin D1/β-Actin expression was similar in both groups (Cinaciguat 0.93 ± 0.10 vs. 1.01 ± 0.14, Figure 7B). The Cyclin D1/p21 ratio increased in lambs treated with Cinaciguat compared to Controls (2.60 ± 0.43 vs. 1.37 ± 0.23, respectively, *p* ≤ 0.05, Figure 7C). The expression of other proteins involved in cell division, such as Phospho-p38 MAPK/p38 MAPK, Phospho-p53/p53, and apoptosis Poly ADP-ribose polymerase (PARP) for the proteolytic fragment (P116-P89) were not different between the two groups (Supplementary Figure S2; Supplementary Table S2).

**FIGURE 8:**
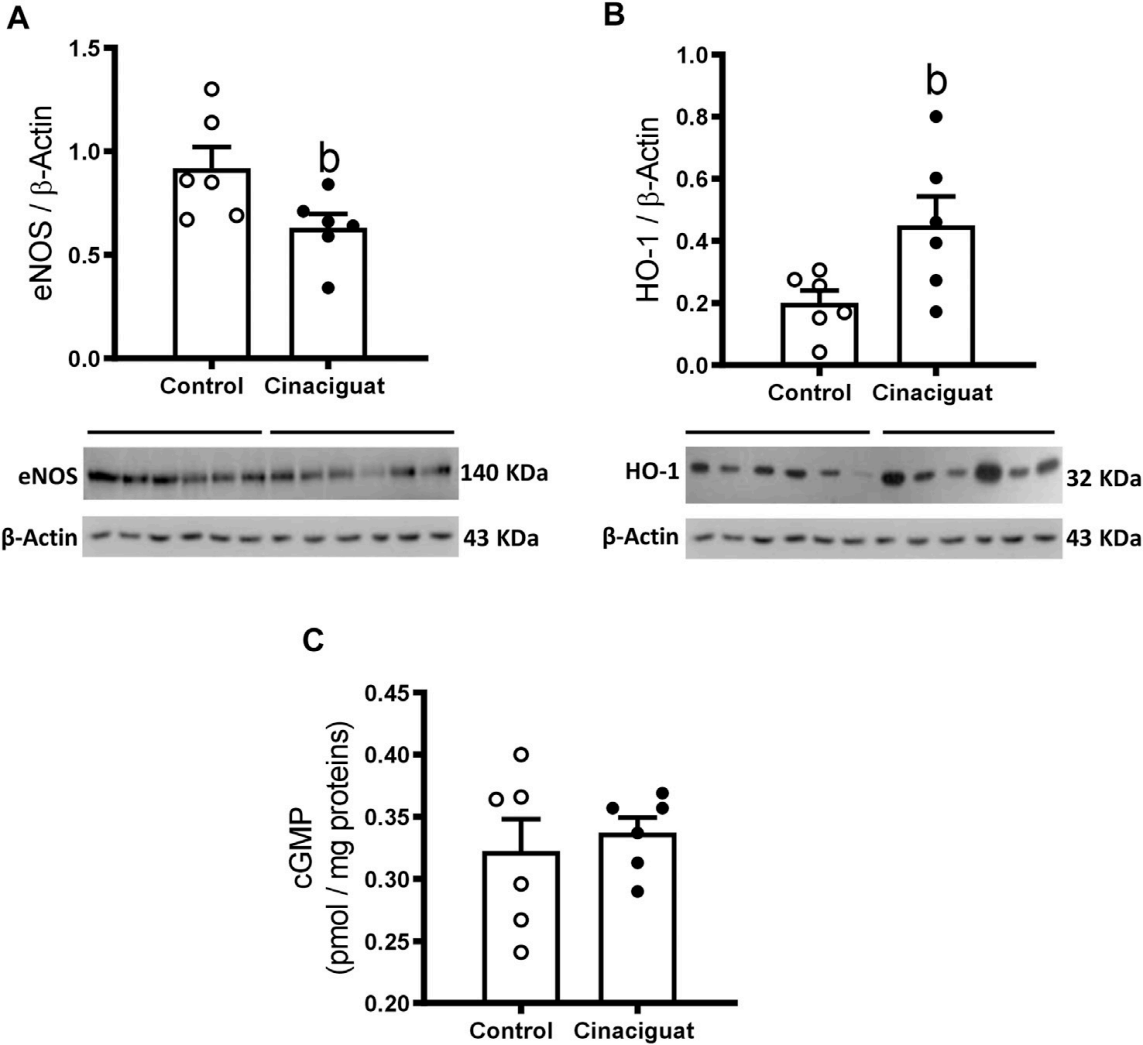
Effect of Cinaciguat treatment on pulmonary expression of vasodilatory pathway proteins and cGMP content in lung tissue in chronically hypoxic and pulmonary hypertensive neonatal lambs. WB of eNOS/β-Actin **(A)**, HO-1/β-Actin **(B)**, and cGMP (pmol/mg proteins) **(C)**. The β-Actin image was re-used for illustrative purposes. Control (Open circles, *n* = 6) and Cinaciguat-treated lambs (Black circles, *n* = 6). Values are means ± SEM. Significant difference (*p* ≤ 0.05): b vs. Control group.

**FIGURE 9:**
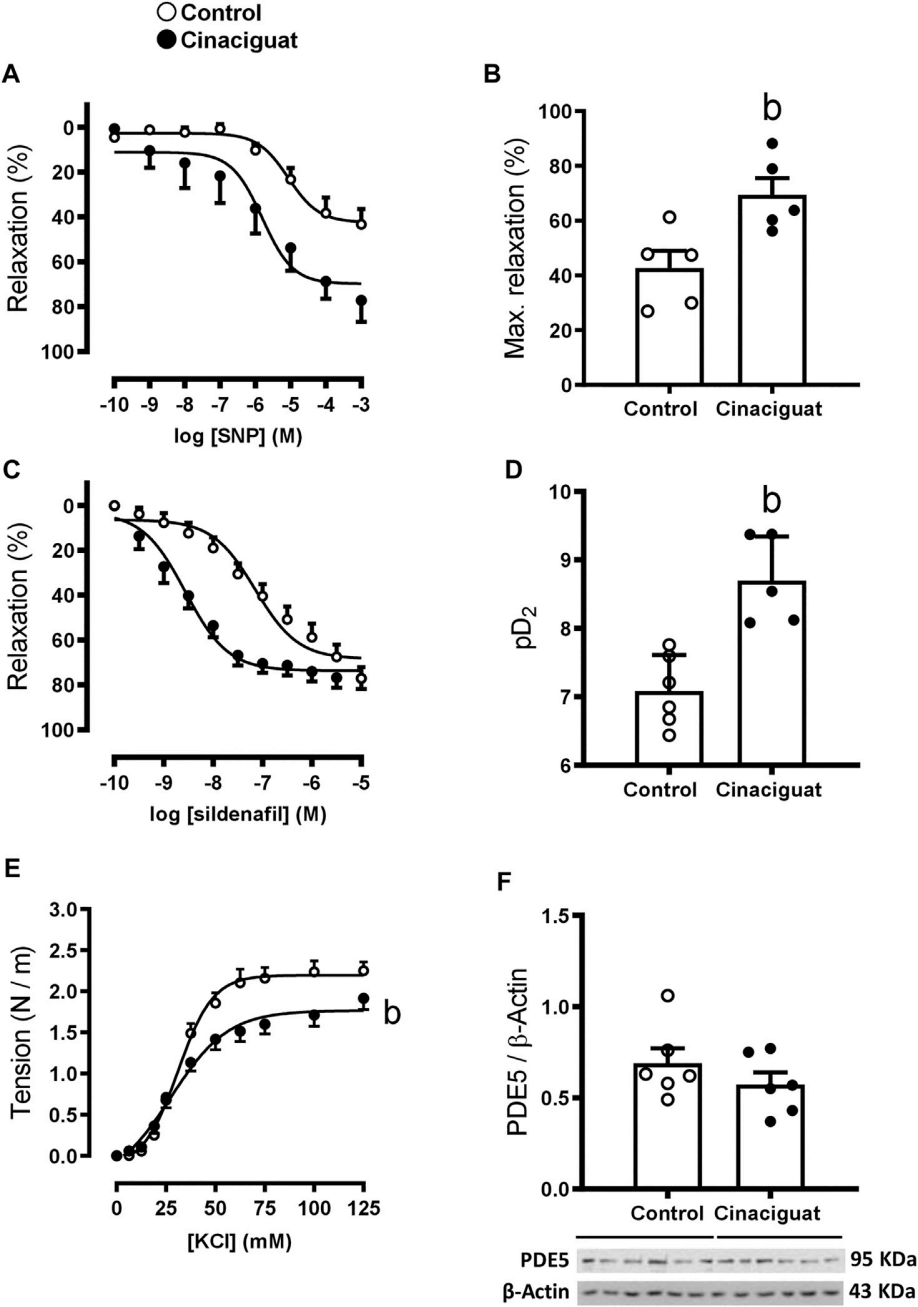
Effect of Cinaciguat treatment on the vasodilator and vasoconstrictor function in isolated small pulmonary arteries and lung protein expression of PDE5 in chronically hypoxic and pulmonary hypertensive neonatal lamb. Responses to SNP and sildenafil of small pulmonary arteries. Y-axis, percent relaxation (%); X-axis, log SNP and sildenafil molar concentration (M) [**(A,C)**, respectively]. Histograms show maximal relaxation (%) and sensitivity (pD2) [**(B,D)**, respectively]. Responses to KCl, Y-axis tension (N/m), X-axis KCl concentration (mM) **(E)**. Western Blot of PDE5/β-Actin **(F)**. The β-Actin image was re-used for illustrative purposes. For SNP experiments, Control (Open circles, *n* = 5) and Cinaciguat-treated lambs (Black circles, *n* = 5). For sildenafil experiments, Control (Open circles, *n* = 6) and Cinaciguat-treated lambs (Black circles, *n* = 5). For KCl experiments, Control (Open circles, *n* = 6) and Cinaciguat-treated lambs (Black circles, *n* = 6). For PDE5 WB experiments, Control (Open circles, *n* = 6) and Cinaciguat-treated lambs (Black circles, *n* = 6). Values are means ± SEM for all experiments. Significant difference (*p* ≤ 0.05): b vs. Control group.

**FIGURE 10:**
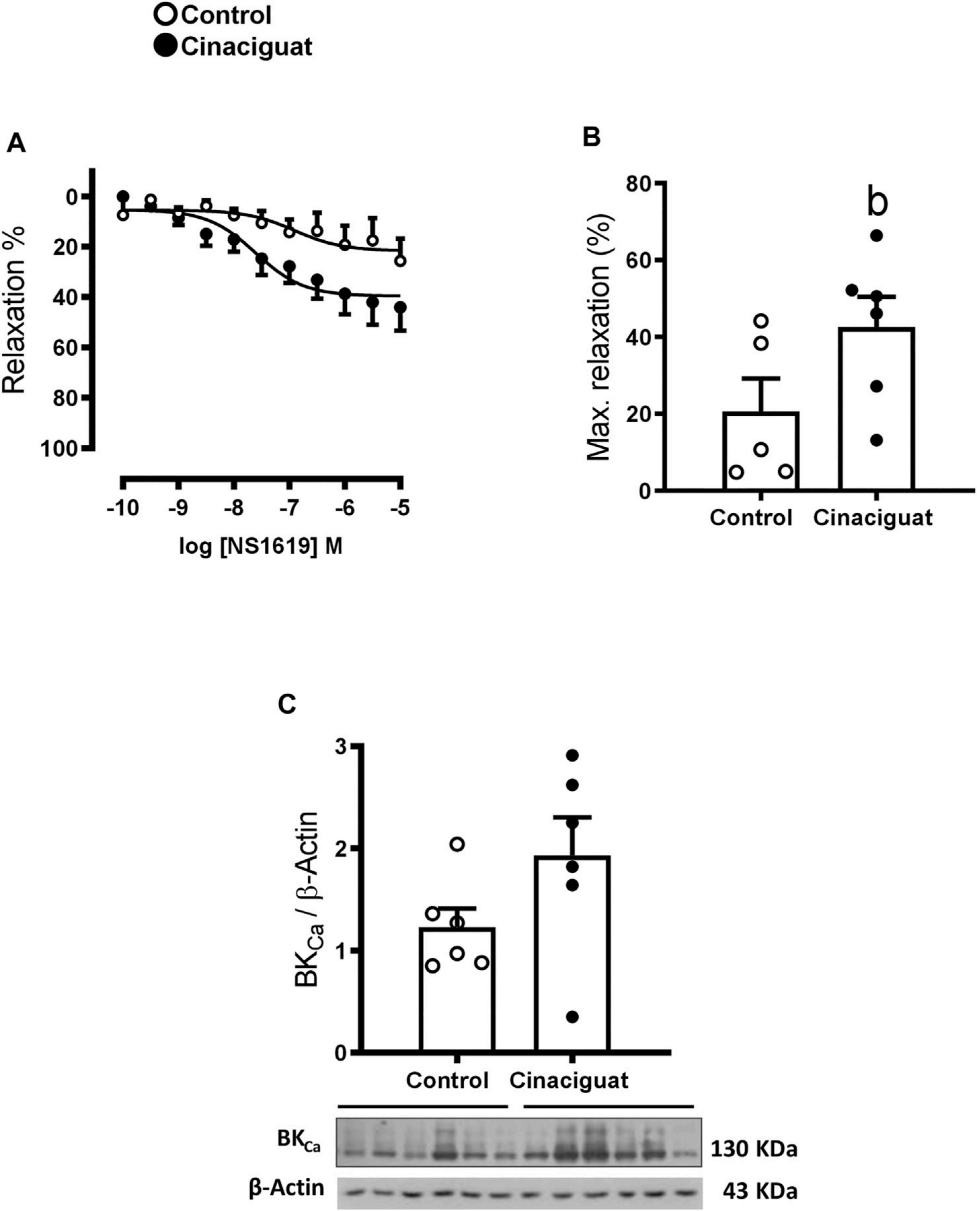
Effect of Cinaciguat treatment on vasodilator function in isolated small pulmonary arteries and lung tissue BK_Ca_ protein expression in chronically hypoxic and pulmonary hypertensive neonatal lambs. Responses to NS1619, BK_Ca_ channel stimulator, in small pulmonary arteries, Y-axis show relaxation (%), X-axis log NS1619 M concentration (M) **(A)**. Histogram show maximal relaxation (%) **(B)**. WB of BK_Ca_/β-Actin **(C)**. The β-Actin image was re-used for illustrative purposes. For NS1619 studies, Control (Open circles, *n* = 5) and Cinaciguat-treated lambs (Black circles, *n* = 6). For BKCa WB, Control (Open circles, *n* = 6) and Cinaciguat-treated lambs (Black circles, *n* = 6). Values are means ± SEM. Significant differences (*p* ≤ 0.05): b vs. Control group.

We assessed the effects of Cinaciguat on the protein levels of the NO-HO-sGC-cGMP-PKG-1-MLC20 pathway. There was an important decrease in eNOS/β-Actin (Cinaciguat 0.55 ± 0.07 vs. Control 0.96 ± 0.14, *p* ≤ 0.05, Figure 8A) and an increase in HO-1/β-Actin (Cinaciguat 0.45 ± 0.09 vs. Control 0.20 ± 0.04, *p* ≤ 0.05, Figure 8B), two important upstream activators of sGC. BK_Ca_/β-Actin protein expression increased in the Cinaciguat group compared to the Control group (2.25 ± 0.24 vs. 1.23 ± 0.18, respectively) but did not reach statistically significance (*p* = 0.089, Figure 10C). In contrast, there were no differences in the cGMP concentration in the lung (Figure 8C), nor in sGC α1/β-Actin and sGC β1/β-Actin protein expression (Supplementary Figure S3; Supplementary Table S3). In addition, PDE5/β-Actin protein expression was similar in both groups (0.57 ± 0.07 Cinaciguat group vs. 0.69 ± 0.08 Control group, Figure 9E). Further, there were no differences in the protein expression of downstream effectors such as PKG-1/β-Actin or MLC20/β-Actin (Supplementary Figure S3; Supplementary Table S3).

Measurements at the *ex vivo* functional level by wire myography demonstrated differences between the responses of small pulmonary arteries of Cinaciguat treated and Control newborns. Keeping in line with the morphological changes shown in Figure 5, the maximal contraction response to KCl was lower in small pulmonary arteries from Cinaciguat than Control newborns (1.71 ± 0.60 (N/m) vs. 2.18 ± 0.08 (N/m), respectively, *p* ≤ 0.05, Figure 9E). In contrast, there was no difference in KCl sensitivity between both groups (22.92 ± 10.28 EC50 (mM) Cinaciguat vs. 31.14 ± 1.23 EC50 (mM) Control). Cinaciguat treatment increased maximal relaxation in response to NO donor SNP vs. Controls (69.49 ± 6.06% vs. 42.70 ± 6.33%, respectively, *p* ≤ 0.05, Figures 9A,B), without affecting sensitivity (pD2) to SNP (5.81 ± 0.31 Cinaciguat group vs. 5.07 ± 0.20 Control group), suggesting increased sGC responsiveness to NO. In contrast, maximal relaxation to sildenafil, a phosphodiesterase five inhibitor, was maintained (73.64 ± 2.11% Cinaciguat group vs. 71.08 ± 2.80% Control group, Figure 9C), but sensitivity (pD2) to sildenafil increased (8.70 ± 0.29 Cinaciguat group vs. 7.10 ± 0.18 Control group, *p* ≤ 0.05, Figure 9D), suggesting increased cGMP catabolism. Finally, there was no change with the protein expression of BK_Ca_ (Figure 10C), but Cinaciguat increased maximal relaxation in the presence of the agonist NS1619 (42.58 ± 7.83% vs. 20.61 ± 8.54%, Cinaciguat and Control lambs, respectively, *p* ≤ 0.05, Figures 10A,B), without changing sensitivity (pD2) to NS1619 (7.61 ± 0.27 vs. 6.91 ± 0.54, Cinaciguat and Control group, respectively).

In summary, these data show that Cinaciguat has important effects on the pulmonary vasculature, altering the arterial number, changing the structure and function of SPA, and altering the expression of several proteins related to cell proliferation and the vasodilatory pathway.

## Discussion

We administered daily pulses of Cinaciguat (BAY-582667), an sGC activator, to chronically hypoxic pulmonary hypertensive newborn lambs. As hypothesized, Cinaciguat treatment reduced pulmonary vascular resistance and decreased right ventricular hypertrophy. Cinaciguat treatment generated these cardiovascular changes through pulmonary circulation remodeling. Lung pulmonary artery density increased, and muscularity-adventitia in pulmonary arteries and arteriole vasodilator tone decreased. Our results show that Cinaciguat has major effects on the right side of the heart and pulmonary circulation.

### Cinaciguat’s Effects on the Cardiovascular System

Cinaciguat (BAY-582667) administration to chronically hypoxic pulmonary hypertensive neonatal lambs had acute and chronic effects on their neonate’s cardiovascular system. Every daily infusion acutely decreased mean pulmonary arterial pressure and pulmonary vascular resistance for approximately 45 min. These fast responses were produced by Cinaciguat-induced sGC activation. sGC activation generates cGMP, which stimulates PKG, phosphorylates MLCP, dephosphorylates MLC, and induces vasodilation (Gao and Raj, 2010). However, cGMP (Van Haastert et al., 1982; Fullerton and McIntyre, 1996; Held and Dostmann, 2012) and Cinaciguat (Frey et al., 2008) have short half-lives; therefore, the effect’s duration was limited. Additionally, Cinaciguat treatment generated a small but significant decrease in mean systemic arterial pressure and resistance and induced tachycardia that persisted for more than 60 min but did not affect cardiac output. The tachycardia could have been caused by a baroreflex induced by the systemic pressure drop (Dampney, 2016; Guyton and Hall, 2016). Cinaciguat also induces noradrenaline release in human normoxic volunteers (Frey et al., 2008). However, the stimulus that generated the tachycardia did not increase cardiac output in the animals treated with Cinaciguat. Control newborns infused with vehicle did not display changes in any of the variables studied (data not shown). Recently, Hung et al., 2021, reported that administration in term newborn rats (10 mg/kg, i.p. at birth) prevented the closure Cinaciguat of the ductus arteriosus, which normally occurs around 2 h after birth in rats. Similarly, hypobaric hypoxia at high altitude also delays ductus arteriosus closure in human neonates (Alzamora-Castro et al., 1960; Penaloza et al., 1964). However, we did not find signs of an open *d. arteriosus* in newborns treated with Cinaciguat. We placed a Swan-Ganz in the pulmonary artery of neonatal lambs on postnatal day 3 and gave a lower daily dose of (35 μg/kg x day, i.v. for 7 days: 245 μg/kg total) from postnatal day 4–10 Cinaciguat than that used by Hung et al., 2021 in newborn rats. Cinaciguat treatment induced an acute decrease in pulmonary arterial pressure for 45 min, which then leveled off until the next dose of Cinaciguat the following day. The presence of a left-to-right shunt through the ductus should increase pulmonary artery pressure and pulmonary artery hemoglobin saturation. However, none of these variables significantly differed from Controls. Furthermore, on day 12, postmortem examination revealed that the ductus arteriosus was closed. Therefore, a small daily dose of Cinaciguat did not modify newborns’ pulmonary arterial pressure and blood gases milieu.

Unexpectedly, after seven daily treatments with Cinaciguat, pulmonary artery pressure in treated newborns was as high as Controls. However, the mechanisms maintaining pulmonary hypertension were different for each group of newborns. Control newborns exhibited a typical decrease in cardiac output with age and PVR continuation (Gonzaléz-Candia et al., 2021). Conversely, PVR decreased in Cinaciguat treated newborns, but they maintained their cardiac output. Therefore, experimental newborns had about 10% higher CO and 10% lower PVR than Controls during the final days of Cinaciguat treatment, but both groups had similar heart rates. Cinaciguat treated newborns maintained a high cardiac output throughout the treatment course, suggesting daily Cinaciguat treatment affects the heart by mediating catecholamine release (Frey et al., 2008). Additionally, as mentioned above, Cinaciguat has direct effects through cGMP, namely *via* PKG-1 mediated titin phosphorylation (Hamdani and Paulus, 2013), increased distension of cardiac cells, and higher ventricular filling. These effects enhance contractile force (Starling’s law) and cardiac output. The lower PVR of Cinaciguat treated newborns suggests important changes in the lung vasculature. The profound differences between Control and Cinaciguat treated newborns were displayed further when lambs were exposed to an episode of acute hypoxia, which was superimposed on an already chronically hypoxic environment. Acute hypoxia increases pulmonary pressure, PVR, cardiac output and heart rate in Control lambs (Herrera et al., 2007). However, acute hypoxia did not increase PVR in Cinaciguat treated newborns, and the response of other variables was blunted, highlighting a significant effect on the pulmonary vasculature. These results are consistent with lesser muscle and adventitia areas in small pulmonary arteries and a potassium induced contraction decrease seen in wire myography, *ex vivo*. The major decrease in pulmonary vascular resistance is also consistent with the increased pulmonary artery density we observed in the lung. These aspects will be discussed later. Cinaciguat treated lambs had lighter right ventricles relative to total lamb weight and lower right ventricle hypertrophic index scores (RVHI). These effects could be caused by decreased pulmonary vascular afterload. However, we cannot exclude the possibility that Cinaciguat acts on the heart directly. Cinaciguat stimulates sGC, generates cGMP and activates PKG. PKG is a kinase that phosphorylates several intracellular proteins that reduce the intracellular calcium concentration, which would decrease the weight of the right ventricle (Francis et al., 2010). Cinaciguat’s effects in the presence of an increased afterload have also been shown in normoxic adult rats subjected to abdominal aortic banding, which causes left ventricular hypertrophy. Aortic banding maintenance did not change systemic blood pressure, but Cinaciguat decreased myocardial hypertrophy in the left side of the heart and increased cardiac contractility (Németh et al., 2016). Using a similar experimental preparation, Cinaciguat administration increased PKG activity and reduced interstitial fibrosis, apoptosis, and nitro-oxidative stress in the left ventricle (Ruppert et al., 2019). Additionally, Cinaciguat inhibits cardiomyocyte hypertrophy *in vitro* (Irvine et al., 2012). Oxidative stress is a common cause of cardiac hypertrophy in several pathological conditions (Ramachandra et al., 2021). These experiments demonstrate that Cinaciguat could reduce left ventricle hypertrophy, despite the high overload suggesting it may also act on the right ventricle. However, we did not observe changes in the expression of proteins related to cell division (Phospho-p38 MAPK/p38 MAPK, Phospho-p53/p53, and Cyclin D1/β-Actin) or apoptosis (PARP) (Supplementary Figure S1; Supplementary Table S1). Nevertheless, we and others (Irvine et al., 2012; Németh et al., 2016; Ruppert et al., 2019) demonstrated that chronic enhancement of cGMP signaling by pharmacological activation of sGC might be a novel therapeutic approach to prevent pathologic myocardial hypertrophy. Future studies are needed to investigate how Cinaciguat decreases right ventricle weight.

### Cinaciguat’s Effects on Pulmonary Arteries

Chronic hypoxia causes pathological pulmonary circulation remodeling. It decreases the lumen of pulmonary arteries, including small pulmonary arteries (SPA), by increasing the smooth muscle layer. It also stimulates cell migration (myofibroblasts, macrophages) from the adventitia to the muscular layer, which increases vessel wall thickness (Stenmark et al., 2018), and increases the expression of extracellular matrix proteins in the adventitia (Gilkes et al., 2013). These remodeled vessels show enhanced contractility *in vitro*, contributing to the increased persistent pulmonary vascular resistance seen in pulmonary hypertension (Stenmark et al., 2006; Penaloza and Arias-Stella, 2007; Gao and Raj, 2011; Stenmark et al., 2018). Chronic hypoxia activates genes related to vascular proliferation (NHE1, TRPC 1,6) and vascular contraction (ET-1, TRPC 1/6, K_v_1.5, and K_v_2.1 channels) through HIF-1 (Prabhakar and Semenza, 2012). Additionally, hypoxia increases reactive oxygen species (ROS) levels in pulmonary tissue. For example, intracellular ROS levels are elevated in pulmonary artery endothelial cells (PAEC) from fetal sheep with a congenital diaphragmatic hernia and pulmonary hypertension and adult rats subjected to hypoxia (Grishko et al., 2001; Acker et al., 2013; Acker et al., 2015), which could induce pulmonary artery remodeling.

We found that Cinaciguat treatment produces major changes in lung vasculature, such as increasing the density of pulmonary arteries, in hypoxic hypertensive newborn lambs. Astorga et al., 2018 reported a similar increase in hypoxic hypertensive lambs treated with the antioxidant hormone melatonin. They found that nitrotyrosine levels, a marker of oxidative stress, were lower in small pulmonary arteries after 10 days of treatment with melatonin. Lung vasculature increased after treatment with sildenafil in a model of fetal pulmonary arterial hypertension generated by constriction of the ductus arteriosus. Additionally, sildenafil and Cinaciguat increased the formation of endothelial cell tubes in pulmonary artery endothelial cells from these fetal lambs *in vitro*, suggesting sGC activation increases angiogenesis (Sharma et al., 2021). Furthermore, superoxide dismutase plus catalase increased tube formation in cultured pulmonary artery endothelial cells by decreasing intracellular ROS (Acker et al., 2015), suggesting ROS inhibits vasculogenesis. Cinaciguat also decrease ROS production *via* PKG-induced antioxidant gene expression in bone osteoblasts from diabetic mice (Kalyanaraman et al., 2018). Similarly, Cinaciguat inhibits mitochondrial ROS elicited by Ang II in ductus arteriosus smooth muscle cells (Hung et al., 2021). We found increased HO-1 protein expression in the lung tissue of lambs treated with Cinaciguat. HO-1 is an enzyme that catalyzes the formation of biliverdin and bilirubin, which are powerful antioxidants that may decrease ROS in the lung. Altogether these data suggest Cinaciguat plays an important role in oxidative stress.

Cinaciguat changed small pulmonary artery structure by decreasing the muscular and adventitia layers in chronically hypoxic pulmonary hypertensive lambs. Similar to our findings with Cinaciguat, treatment with 2-APB (a store-operated channel blocker) (Castillo-Galán et al., 2016), Fasudil (a Rho-kinase inhibitor) (Lopez et al., 2016), hemin (a hemoxigenase (HO-1) stimulator) (Ebensperger, 2011), and melatonin (a potent antioxidant) (Astorga et al., 2018), decreased muscle area. Additionally, three of these studies observed decreased smooth muscle cell proliferation, determined using Ki67. Conversely, we found newborns treated with Cinaciguat had a thinner muscular layer containing more putative smooth muscle cells. We observed more Ki67 positive arteries in experimental groups than Controls, indicating this effect was due to increased proliferation and did not observe any changes in the apoptosis markers (PARP and mRNA of Bax/Bcl2). Additionally, the lung tissue of experimental lambs had a higher Cyclin D1/p21 ratio, indicating increased cell proliferation. The consensus is that cGMP prevents vascular smooth muscle cell (VSMC) proliferation. However, new studies examining the effect of sustained NO-cGMP-PKG axis activation on VSMC growth and angiogenesis have challenged this consensus (Lehners et al., 2018). Some *in vitro* studies suggest that NO-cGMP-PKG pathway stimulation increases cell proliferation in aortic smooth muscle cells from normoxic adult rats and mice (Hassid et al., 1994). In normoxic mice, 8-Br-cGMP, a cGMP analogue, and low levels of NO increase proliferation mechanisms in smooth muscle cells (Wolfsgruber et al., 2003). We observed decreased expression of eNOS protein in the lungs of our chronically hypoxic Cinaciguat treated lambs accompanied by proliferative pathway activation in small pulmonary arteries. We also observed a higher cell density in the SPA muscle layer. We hypothesize that Cinaciguat reduces cell size. Cinaciguat activates BK_Ca_ *via* PKG-1 phosphorylation in cultures of smooth muscle cells from human corneal sclera. BK_Ca_ activation triggered the efflux of potassium, chloride, and water into the extracellular space and reduced smooth muscle cell size (Dismuke et al., 2009; Ellis, 2011). We did not directly investigate whether the same mechanism decreased smooth muscle cell size in our experiments.

We tested Cinaciguat’s effects on small pulmonary arterial function *ex vivo via* myography. As mentioned above, Cinaciguat treatment decreased eNOS protein expression and increased HO-1but did not affect PDE5 protein expression and cGMP content compared to Controls.

We assessed pulmonary contractility by examining the response of small pulmonary arteries to KCl and found Cinaciguat treatment decreased small pulmonary artery contractility. Additionally, arteries from Cinaciguat treated lambs had a larger vasodilatation response to sodium nitroprusside (SNP) than arteries from Controls. SNP is an exogenous donor of NO, which only exerts an effect when the sGC enzyme has reduced Fe^2+^ in its heme group (Ataei Ataabadi et al., 2020). sGC α1 and sGC β1 expression in the lung was the same in both groups of neonates; therefore, the greater vasodilatation response to SNP in the myographic experiment supports an increase in sGC function. Cinaciguat reduces oxidative stress (Kalyanaraman et al., 2018; Hung et al., 2021) by increasing iron reduction to Fe^2+^ in sGC and sGC activity. Concurrently, Ebensperger, 2011, found that treating chronically hypoxic and high altitude pulmonary hypertensive animals with hemin increased HO-1 protein expression and SNO induced relaxation compared to Controls. Additionally, increased HO-1 protein expression stimulated CO generation, which stimulated sGC and increased vasodilation (Nachar et al., 2001; Ebensperger, 2011). Another possible explanation for a lower cellular oxidative state was raised by Salloum et al., 2012, who used Cinaciguat as a pre-injury treatment for a ischemia/reperfusion experiment in mouse cardiomyocytes. They found Cinaciguat treatment increased protein and transcript (mRNA) expression of cystathionine-γ-lyase, an enzyme that generates hydrogen sulfide (H_2_S). Hydrogen sulfide (H_*2*_S) acts as a ROS scavenger by forming the antioxidant glutathione and increasing activation of enzymes such as HO-1 (Calvert et al., 2009), which we observed in our study. In conclusion, Cinaciguat affects the cell’s redox state by increasing several molecules with antioxidant effects and reducing intracellular ROS, which is associated with neonatal pulmonary hypertension.

The BK_Ca_ channel is another important vasodilator molecule, and BK_Ca_ protein expression was higher in the lungs of Cinaciguat treated lambs compared to Controls, but not statistically different. However, we found that treatment with NS1619, an activator of the BK_Ca_ channels, increased vasodilation *ex vivo*. The vasodilation was similar to that obtained in rats with pulmonary hypertension treated with NS1619 (Revermann et al., 2014) and pulmonary hypoxic-hypertensive lambs treated with hemin (Ebensperger, 2011). PKG phosphorylates and activates BK_Ca_ channels (Francis et al., 2010), which hyperpolarizes smooth muscle cells. This hyperpolarization blocks voltage-dependent Ca^2+^ channels, decreases intracellular Ca^2+^ concentration and triggers vasorelaxation (Gao and Raj, 2010). Therefore, Cinaciguat treatment improves vasodilation by increasing the function of BK_Ca_ channels.

Phosphodiesterase 5 (PDE5) catabolizes cGMP (Ebensperger, 2011), the starting molecule in a signaling cascade that triggers vasodilation. We generated concentration-response curves with sildenafil (Herrera et al., 2008), a competitive PDE5 inhibitor, to investigate the role of PDE5 in regulating arterial tone and found that sildenafil increased sensitivity (pD2) in Cinaciguat-treated neonatal lambs compared to Controls. This increased sensitivity suggests increased PDE5 function in neonates treated with Cinaciguat, which could occur *via* two mechanisms; phosphorylation of PDE5 by the cyclic GMP-dependent protein kinase-1 (PKG-1) (Rybalkin et al., 2002; Ahmed et al., 2021) and PDE5 activation by cGMP binding to the allosteric sites of PDE-5 (negative feed-back) (Rybalkin et al., 2003; Ahmed et al., 2021). Repeated Cinaciguat doses for 7 days enhanced sGC activation, increased intracellular cGMP concentration, stimulated PKG-1 and increased PDE-5 function. Increased PDE-5 function in the arterioles contributed to potent vasodilation in Cinaciguat-treated neonatal lambs compared to Controls.

A decreased contractile response and increased response to vasodilating agents are consistent with the structural changes observed in the lung vasculature and support the notion that Cinaciguat has significant effects on pulmonary vasculature.

However, a potential limitation of this work is the lack of a lowland Control group.

In summary, Cinaciguat has important effects on the cardiovascular system of chronically hypoxic pulmonary hypertensive neonates. It lowers pulmonary vascular resistance and decreases right ventricle hypertrophy. These effects are closely related to Cinaciguat’s major effects on pulmonary vasculature. Cinaciguat remodels and alters the function of pulmonary arterioles, decreases basal pulmonary vascular resistance, and reduces cardiovascular responses following a superimposed episode of acute hypoxia in neonatal lambs. Therefore, Cinaciguat could be a useful therapeutic tool to reduce pulmonary vascular remodeling and/or right ventricular hypertrophy in neonatal pulmonary hypertension.

## Data Availability

The original contributions presented in the study are included in the article/Supplementary Material, further inquiries can be directed to the corresponding author.
