# Supplementary figures and images for: Cinaciguat (BAY-582667) Modifies Cardiopulmonary and Systemic Circulation in Chronically Hypoxic and Pulmonary Hypertensive Neonatal Lambs in the Alto Andino
Source: Front Physiol. 2022 Jun 6;13:864010. doi: 10.3389/fphys.2022.864010 (PMC9207417; doi:10.3389/fphys.2022.864010)

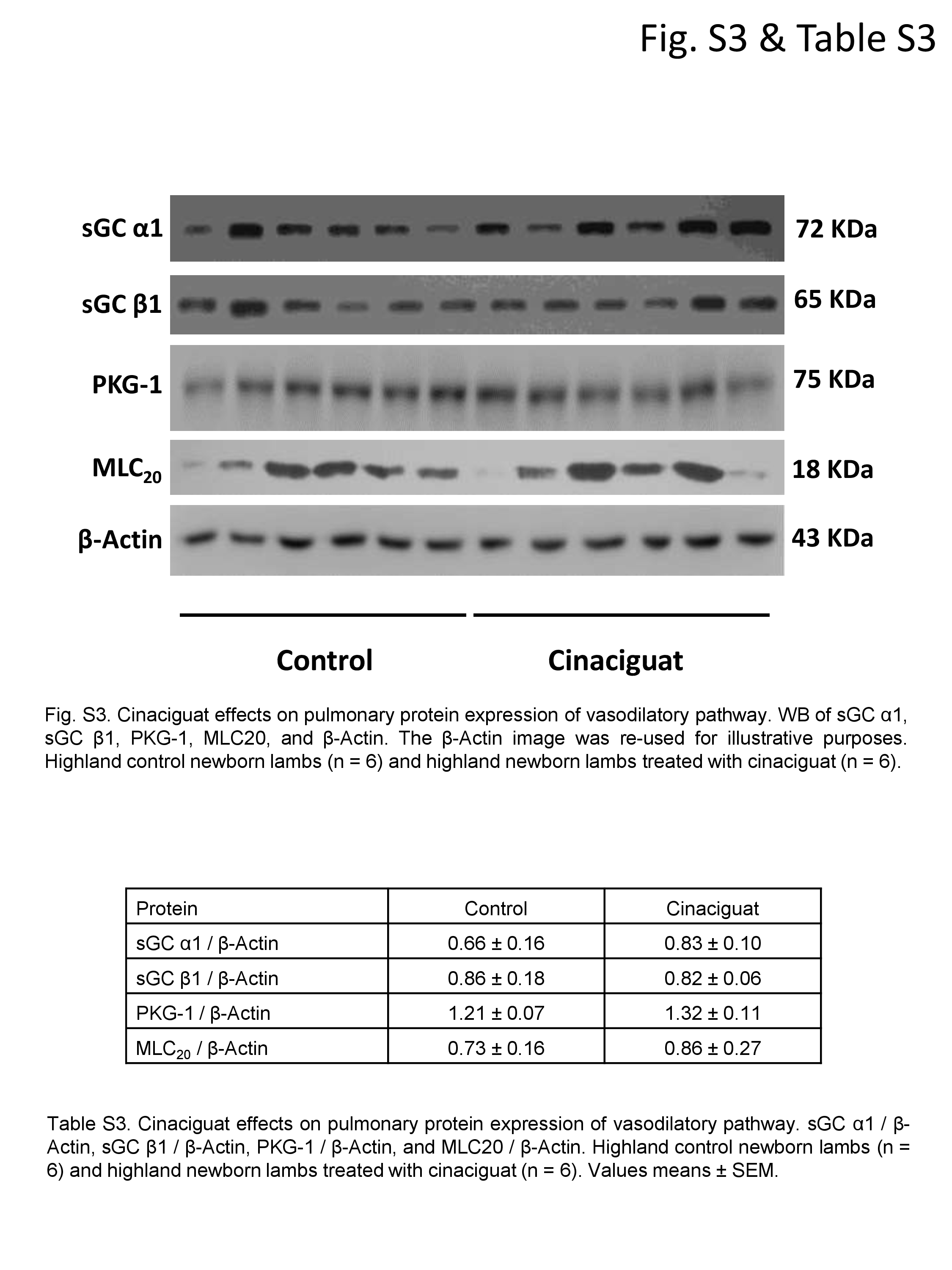

Supplement: Supplementary file 1 [file Image3.tiff]

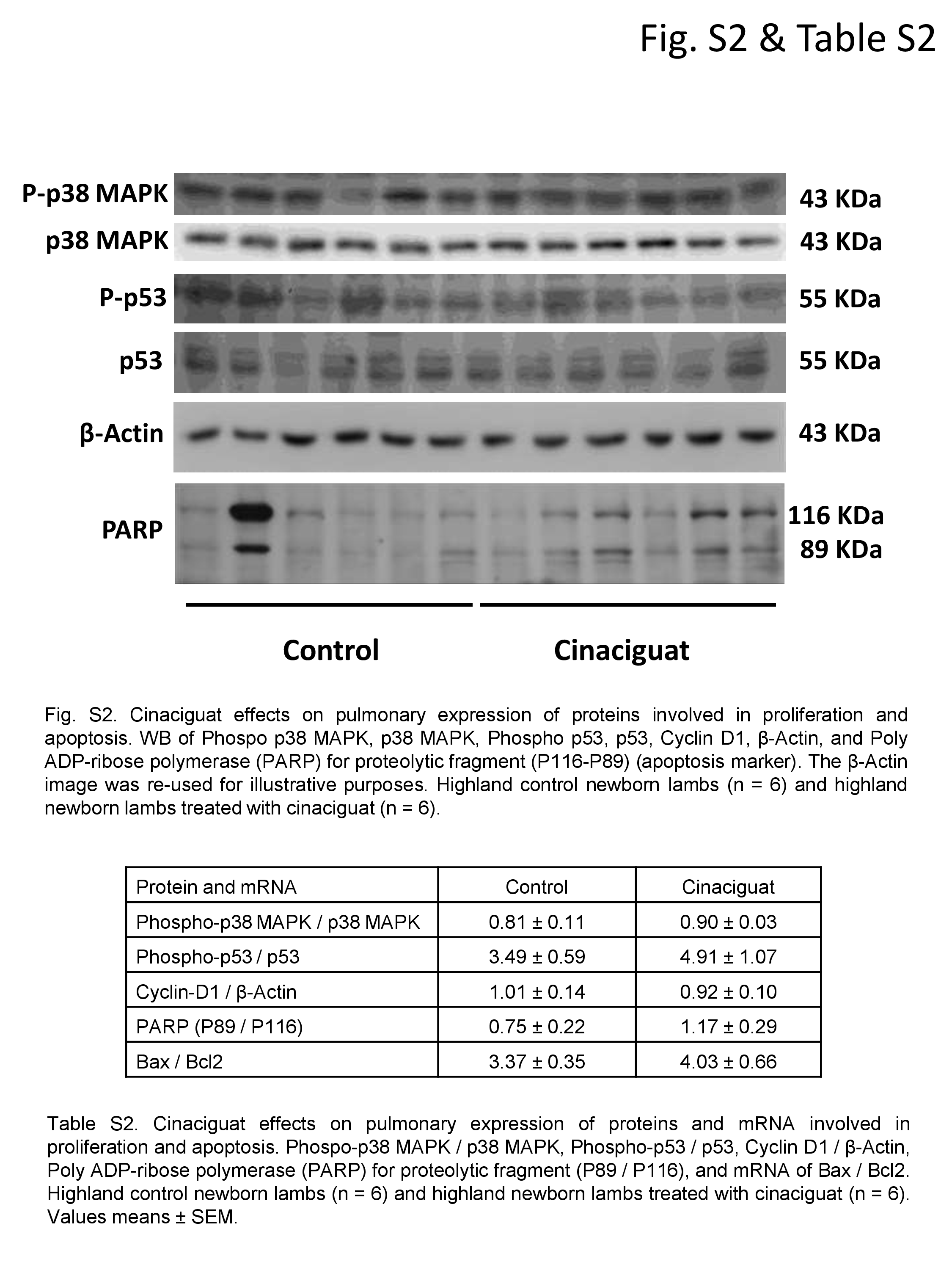

Supplement: Supplementary file 3 [file Image2.tiff]
